# The production of l- and d-phenylalanines using engineered phenylalanine ammonia lyases from *Petroselinum crispum*

**DOI:** 10.1038/s41598-019-56554-0

**Published:** 2019-12-27

**Authors:** Souad Diana Tork, Emma Zsófia Aletta Nagy, Lilla Cserepes, Diana Monica Bordea, Botond Nagy, Monica Ioana Toşa, Csaba Paizs, László Csaba Bencze

**Affiliations:** Biocatalysis and Biotransformation Research Center, Faculty of Chemistry and Chemical Engineering, Babeș-Bolyai, University of Cluj-Napoca, Arany János Str. 11, RO-400028 Cluj-Napoca, Romania

**Keywords:** Biocatalysis, Biocatalysis

## Abstract

The biocatalytic synthesis of l- and d-phenylalanine analogues of high synthetic value have been developed using as biocatalysts mutant variants of phenylalanine ammonia lyase from *Petroselinum crispum* (*Pc*PAL), specifically tailored towards mono-substituted phenylalanine and cinnamic acid substrates. The catalytic performance of the engineered *Pc*PAL variants was optimized within the ammonia elimination and ammonia addition reactions, focusing on the effect of substrate concentration, biocatalyst:substrate ratio, reaction buffer and reaction time, on the conversion and enantiomeric excess values. The optimal conditions provided an efficient preparative scale biocatalytic procedure of valuable phenylalanines, such as (*S*)-*m*-methoxyphenylalanine (Y = 40%, ee > 99%), (*S*)-*p*-bromophenylalanine (Y = 82%, ee > 99%), (*S*)-*m*-(trifluoromethyl)phenylalanine (Y = 26%, ee > 99%), (*R*)-*p*-methylphenylalanine, (Y = 49%, ee = 95%) and (*R*)-*m*-(trifluoromethyl)phenylalanine (Y = 34%, ee = 93%).

## Introduction

Substituted phenylalanines are important chiral building blocks for pharmaceutical industry, their incorporation into small-molecule therapeutic agents, or peptides and proteins, is commonly approached^1,2^. Biocatalytic procedures continuously emerged with the aim to provide green alternatives for the production of these valuable synthons. Phenylalanine ammonia lyases (PALs, EC 4.3.1.24 and PAL/TALs with combined phenylalanine and tyrosine ammonia lyase activities, EC 4.3.1.25) are among the most studied biocatalysts for the production of optically pure d- and l-phenylalanine analogues^3–14^ with PAL-based industrial processes already known, such as the multitone scale production of (*S*)-2,3-dihydro-1*H*-indole-2-carboxylic acid by DSM (Netherlands)^15^. The phenylalanine ammonia lyase from *Petroselinum crispum* (*Pc*PAL) is one of the most intensively employed PALs, that either as whole cell, purified or immobilized biocatalyst, provided a wide range of both l- and d-phenylalanine analogues^16–21^. Protein engineering of PALs^8,9,12,22–25^, but also of the structurally and mechanistically closely related phenylalanine aminomutases (PAMs, EC 5.4.3.10/11)^26–29^, with mixed α- and β-regioselectivity^30,31^, provided variants with enhanced activity/selectivity towards several arylalanine derivatives. Recently the mapping of the hydrophobic binding region of *Pc*PAL active site^8^ revealed that its catalytic efficiency towards targeted substrates can be tailored through rational design, considering the position (*ortho-, meta-* or *para-*) of substrate’s ring-substituent.

**Figure 1:**
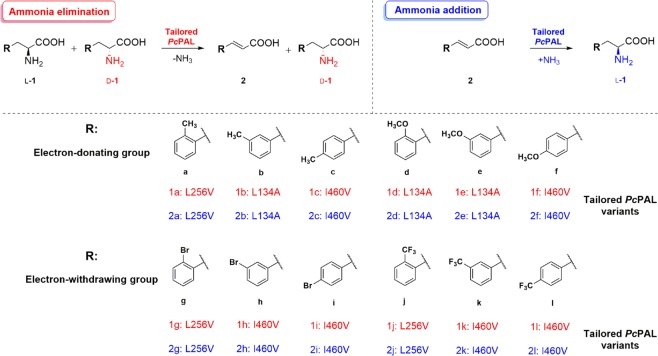
The ammonia addition and ammonia elimination reactions of ring-substituted cinnamic acids and racemic phenylalanines, catalyzed by the corresponding tailored *Pc*PAL variant^8^.

Based on these results, within this study we focused on the development of efficient biocatalytic technologies for the production of several valuable l- and d-Phe analogues, using the specifically tailored *Pc*PAL variants^8^ (Fig. 1). The targeted Phe-analogues include l-*p*-bromophenylalanine l-**1i**, an important intermediate in the production of several biarylalanines^12^ or l-*m*-(trifluoromethyl)phenylalanine l-**1k**, integrated in kinesin KIFC1 inhibitors^32^, with essential role in centrosomal bundling within cancer cells. Similarly, its enantiomer pair d-*m*-(trifluoromethyl)phenylalanine d-**1k** is key chiral intermediate for (*R*)-PFI-2^33^, a potent inhibitor for SET domain containing lysine methyltransferase 7 (SETD 7) involved in multiple cancer-related signaling pathways, while d-*p*-methylphenylalanine d-**1c** is incorporated into Pin1 inhibitors^34^, as well as anti-inflammatory formyl peptide receptor 1 antagonist^35^. Methoxy-substituted phenylalanines, such as l-*p*-methoxyphenylalanine l-**1f** and l-*m*-methoxyphenylalanine l-**1e** are also well-known key intermediates for the synthesis of tamsulosin^36^ and HIV protease inhibitors^37^, respectively.

## Results and Discussion

While *Pc*PAL in terms of catalytic efficiency (k_cat_, k_cat_/K_M_) is among the most efficient PALs both for the natural ammonia elimination and for the reverse ammonia addition reactions^10,38^, its tailored mutant variants, in comparison with the wild-type enzyme, present even superior kinetic parameters within the reactions of the non-natural substrates^8^ (Fig. 1). Accordingly, in order to develop efficient preparative scale PAL-procedures towards phenylalanines of high synthetic interest, we focused on the reaction engineering of both ammonia addition and ammonia elimination reaction routes (Fig. 1), using as biocatalysts the engineered *Pc*PAL variants.

Notable, that the ammonia addition reactions, as asymmetric synthetic procedure present advantageous synthetic potential over the ammonia elimination reactions: 100% theoretical yield for l-Phe analogues and the use of synthetically accessible, achiral starting materials as compared to the kinetic resolution type ammonia eliminations, that yields the d-phenylalanines in maximal theoretical yield of 50% from their racemic mixtures. Higher yields of d-phenylalanines can be obtained by coupling the ammonia addition reaction with a stereoselective oxidation catalyzed by L-amino acid deaminases, followed by a chemical non-selective reduction step, within a one-pot procedure^39^.

### Optimization of ammonia additions

During the optimizations of ammonia addition reactions, we studied the effect of the reaction medium/ammonia source, biocatalysts:substrate ratio, substrate concentration on the conversion and enantiomeric excess (*ee*) values, using *p*-methylcinnamic acid **2c** and *p*-(trifluoromethyl)cinnamic acid **2l** as model substrates.

#### The effect of ammonia source

**Figure 2:**
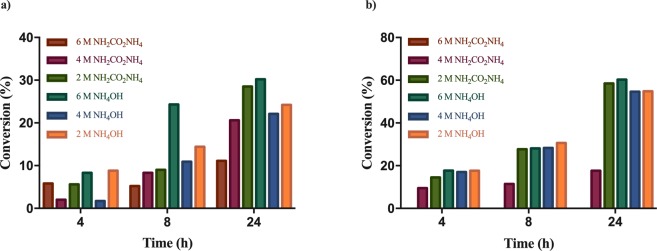
Conversion values of ammonia addition reactions of (**a**) *p*-CH_3_-cinnamic acid **(c**,**b)** *p*-CF_3_-cinnamic acid **2l**, using different ammonia sources. In all points of the reactions the enantiomeric excess (*ee*) values were >99%, while in case of using 6 M NH_4_[H_2_NCO_2_] no conversion of **2l** could be detected.

The effect of the reaction medium, serving both as ammonia source and reaction buffer, was tested at 2 mM **2c** and **2l** using whole cell *Pc*PAL-biocatalysts in cell densities of OD_600_ ~ 1 (~6 mg wet cells/mL) in aqueous solutions of different concentrations of ammonia (2, 4, 6 M NH_4_OH pH 10 adjusted with CO_2_) or ammonium carbamate (2, 4, 6 M NH_4_[H_2_NCO_2_], pH 9.6–10 without adjustment) (Fig. 2a,b). While in previous PAL-mediated biotransformations 2 M and 4 M ammonium carbamate proved to be the optimal ammonia source^11,24^, in our case the highest conversions were achieved using 6 M NH_4_OH (30.2% for **2c** and 60.3% for **2l** after 24 h), while using high ammonium carbamate concentrations (4–6 M) significantly lower conversion values (11.1%, 20.6% for **2c** and <1%, 17.6% for **2l** after 24 h, respectively) were obtained. In accordance with the fact that ammonium carbamate can provide 2 molecules of ammonia, instead of 1 provided by NH_4_OH and also an overall lower ionic strength, the conversions using 2 M NH_2_CO_2_NH_4_ approximated the optimal values registered in 6 M NH_4_OH (Fig. 2a,b), but not exceed them as in previous studies^11,24^. Under lower ammonia and ammonium carbamate content significantly decreased conversion values were obtained, supporting the necessity of high ammonia concentration for the reverse, ammonia addition reactions.

#### The effect of biocatalyst:substrate ratio

**Figure 3:**
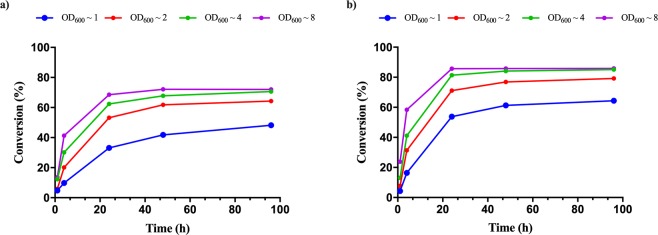
The effect of increased cell densitities of the whole-cell *Pc*PAL biocatalysts upon the conversion values of ammonia addition onto (**a)** *p*-methylcinnamic acid **2c** and (**b)** *p*-(trifluoromethyl)cinnamic acid **2l**. In all points of the reactions the *ee* values were >99%.

The biocatalyst:substrate ratio was varied, by using different densities of whole cell *Pc*PAL biocatalysts (OD_600_ of ~1, 2, 4, 8 corresponding to a wet cell concentration of ~6, 12, 24, 48 mg/mL) at fixed 2 mM concentration of model substrates **2c** and **2l**, monitoring the conversions and *ee* values of products for the corresponding ammonia addition reactions (Fig. 3a,b). While cell densities of OD_600_ of ~4, 8 provided the highest conversions, they significantly enhanced the viscosity of the reaction medium, hindering both sample preparation and reaction monitoring by HPLC (appearance of additional, interfering signals). Thus, cell densities of OD_600_ ~2, corresponding to biocatalyst: substrate ratio (OD_600_: mM) of 1 was selected for further experiments, as they provided relatively high conversions (61.8% for **2c** and 76.9% for **2l**) and moderate medium viscosity for the further optimization steps.

#### The effect of substrate concentration

The solubilities of model substrates **2l** and **2c** were tested in the reaction buffers providing the highest conversions (6 M NH_4_OH and 2 M ammonium carbamate, Fig. 2a,b). Besides the conversion values (Fig. 2a,b), the solubilities of the model substrates **2l** and **2c** were also higher in 6 M NH_4_OH in comparison with the 2 M ammonium carbamate solution, providing substrate concentrations below 5 mM.

Therefore, using the optimal reaction medium (6 M NH_4_OH at pH 10), and maintaining the biocatalysts:substrate concentration ratio (OD_600_: mM) at the optimal value of 1, the ammonia additions onto all substrates **2a-l** were performed varying the substrate concentration in the limit of their solubilities (Figs. 4a,b and S2–S11).

**Figure 4:**
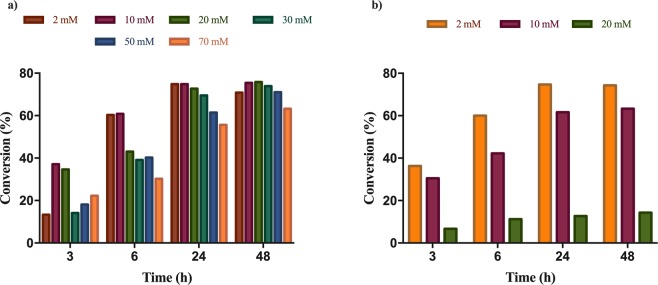
The effect of substrate concentration on the conversion values of ammonia additions onto (**a)** *m*-OCH_3_-cinnamic acid **2e** using L134A *Pc*PAL (**b)** *p*-Br-cinnamic acid **2i** using I460V *Pc*PAL. In all points of the reactions the enantiomeric excess (*ee*) values were above 99%.

In most cases the progression of the conversions slowed down with increasing substrate concentration, leading to longer reaction times at high substrate concentrations.

The *ortho*-substituted substrates **2a,g,j**, were transformed with high conversions of 66–91% after 48 h reaction time, even substrate concentrations as high as 30–70 mM (Figs. S2, S7, S9). In contrast, the ammonia addition onto *o*-OCH_3_-substituted **2d** was seriously affected by the increase of substrate concentrations above 10 mM. In this case the stationary conversions decreased from the moderate ~46% to lower values of ~25%, when using substrate concentrations of 2–10 mM and 20–70 mM, respectively (Fig. S5).

Similar tendency was registered for *meta*-substituted substrates, where concentrations higher than 2 mM substantially decreased the conversion values (Figs. S3, S8, S10 for **2b,h** and **k**), except for *m*-methoxycinnamic acid **2e** (Fig. 4a), where conversion values maintained at ~71% even at high 50 mM substrate concentration.

In case of *para*-substituted substrates, substrate concentrations not affecting the final conversions were higher for substrates with electron donating substituents (30 mM for **2c** and **2f** – Figs. S4, S6) than for substrates with electron withdrawing substituents (10 mM and 2 mM for **2i** and **2l**, respectively - Figs. 4b, S11).

In case of *m*-CH_3_-cinnamic acid **2b**, *o*-OCH_3_-cinnamic acid **2d**, *p*-CF_3_-cinnamic acid **2l** the ammonia additions stopped at low conversions (15–42%) even in case of low substrate concentrations (2–10 mM, Figs. S3, S5, S11), suggesting the occurrence of substrate inhibition. This was further supported by the inhibitory effect of increasing substrate concentration on the reaction velocities of the ammonia addition reaction of **2l**, determined through the UV-based PAL-activity assay, using purified I460V *Pc*PAL as biocatalyst (Fig. S22).

Using the optimal conditions specific for each substrate, the ammonia additions onto all substrates **2a-l** were performed, with the aim to determine the final, stationary conversions and *ee* values. Most of the reactions proceeded with high or moderate conversions and provided the l-Phe derivatives l-**1a-l** in excellent *ee*s (Table 1). As exception, the ammonia addition reactions of **2b** and **2f** stopped at low stationary conversions of 26.4% and 19.1%, respectively (Table 1), which were maintained even after adding fresh batch of *Pc*PAL-whole cells (preceded by the removal of the initial whole cell-batch by centrifugation). The significantly decreased conversions and reaction velocities of the ammonia addition onto **2b** and **2f**, performed in presence of increasing concentrations of **1b** and **1f**, respectively, supports the occurrence of product inhibition in these cases (Figs. S24–S26).

**Table 1:** Conversion and enantiomeric excess (*ee*) values of L-**1a-l** obtained in the ammonia addition reactions performed under optimal conditions^a^.

Substrate	Substituent	PcPAL	[S] (mM)	Time (h)	c (%)	ee_L_ (%)
2a	o-CH_3_	L256V	70	24	91.5	>99
2b	m-CH_3_	L134A	2	48	26.4	>99
2c	p-CH_3_	I460V	30	48	59.9	>99
2d*	o-OCH_3_	L134A	10	24	42.5	>99
2e*	m-OCH_3_	L134A	50	48	71.0	>99
2f*	p-OCH_3_	I460V	30	48	19.1	>99
2g*	o-Br	L256V	70	24	81.6	>99
2h*	m-Br	I460V	5	24	70.1	98.3
2i*	p-Br	I460V	10	24	61.6	>99
2j*	o-CF_3_	L256V	20	24	73.0	>99
2k	m-CF_3_	I460V	2	24	77.5	>99
2l*	p-CF_3_	I460V	2	48	42.2	>99

^a^*Reaction conditions*: assays were performed in 1.5 mL polypropylene tubes at 30 °C, 200 rpm for 16 h, in 500 µL reaction volume, using 2–70 mM substrate concentration, ratio of cell density (OD_600_)/substrate concentration (mM) of 1.0 and 6 M NH_4_OH at pH 10 (adjusted with CO_2_) as reaction medium. *The optimal substrate concentrations were selected considering the shortest reaction time leading to highest *ee* and conversion values.

In terms of comparison with similar PAL mediated procedures, the optimized biotransformations provide superior conversions and enantiomeric excess values for the ammonia additions onto *o-, m-, p*-OCH_3_-cinnamic acids to those reported for the wild-type *Pc*PAL^8^ or PAL from *Anabaena variabilis* (*Av*PAL)^10,40^, and approximates, in case of *o*-OCH_3_-cinnamic acid even surpasses, the conversions obtained with the improved variants of PAL from *Planctomyces brasiliensis* (*Pb*PAL), reported for its remarkable high activity towards cinnamic acids bearing electron donor ring substituents^25,40^. The high enantiomeric excess values of the trifluoromethyl-substituted l-phenylalanines l-**1j**-**k** obtained with moderate to high conversions (Table 1) exceed the ee values previously obtained through wild-type *Av*PAL, *Pc*PAL or PAL from *Rhodotorula glutinis (RgPAL)*^10,39^. In case of *p*-Br-substituted substrate **2i**, the obtained results are comparable with the procedures based on improved *Av*PAL variants^12^, but are surpassed by the conversions obtained by the 31E variant of *Rhodotorula graminis* PAL *(RgrPAL)*^24^. While *Av*PAL-mediated ammonia additions onto methyl-substituted cinnamic acids were also reported^25^, the optimized *Pc*PAL procedures provide superior conversions (in case of *o*- and *p*- methylcinnamic acids) and enantiomeric excess values (for *o*- and *m*-methylphenylalanine) (Table 1).

### Ammonia elimination optimizations

For the optimization process of the kinetic resolution-type ammonia eliminations from racemic phenylalanine analogues, *rac*-**1c** and *rac*-**1k** were chosen as model substrates. Besides aiming to achieve conversion approximating the optimal 50% of a highly selective kinetic resolution, we monitored the enantioselectivity of the resolution process, by the correlation of the experimentally determined *ee* values of the unreacted d-enantiomer with the theoretical *ee* values, calculated from the obtained conversions, considering a fully enantioselective resolution process. Since during a kinetic resolution of high enantioselectivity (with E»200), only the ammonia elimination from the l-Phe derivative occurs, the *ee* of the unreacted d-enantiomer increases upon the progress of the reaction and reaches the optimal value of 100% at conversion values approximating 50%.

#### The effect of reaction medium

The optimal pH of PALs generally ranges from 8.2–9.5^41–43^. In accordance with these data, using whole cell *Pc*PAL biocatalyst in Tris-buffers of different pH values as reaction medium, the highest conversions were obtained at pH values >8.8. (Fig. S1). Further, the influence of various buffer systems, such as Tris (20 mM Tris.HCl, 120 mM NaCl, pH 8.8), NH_3_-buffer (0.1 M NH_4_OH, pH 9.5, adjusted with CO_2_), borax (0.1 M Na_2_[B_4_O_5_(OH)_4_], pH 9.5), ammonium acetate buffer (0.1 M CH_3_COONH_4_, pH 9.5,) sodium carbonate (0.1 M Na_2_CO_3_, pH 9.0) and phosphate-buffer (0.1 M Na_2_HPO_4_-NaH_2_PO_4_, pH 8.8), on the conversion and *ee* values were studied using a fixed substrate concentration of 2 mM from model substrates, *rac*-**1c** and *rac*-**1k**, and a 1:2 whole-cell *Pc*PAL biocatalyst:substrate ratio (OD_600_:mM). The pH values of all tested buffers position within the found optimal pH domain (Fig. S1).

**Figure 5:**
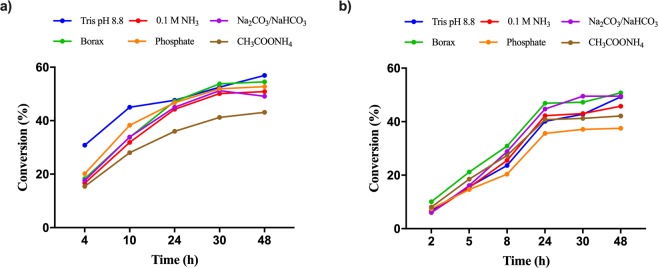
Conversion values of ammonia elimination reactions performed in different buffers, using I460V *Pc*PAL as biocatalyst and **(a)** *rac-m*-CF_3_-phenylalanine *rac*-**1k** or **(b)** *rac-p*-CH_3_-phenylalanine *rac*-**1c** as substrate. In all cases the experimentally determined *ee* values of the nonreacted d-**1k,1c** were in accordance with the theoretical *ee* values, calculated from the corresponding conversion values (data not shown).

In case of *rac-m*-(trifluoromethyl)phenylalanine (*rac*-**1k**), the conversions reached 50% in all cases after 30 h reaction time with the exception of ammonium acetate buffer, where the maximum conversion was not reached even after 48 h (43.1%). The highest conversions and ee (>99%) was obtained using Tris, borax or phosphate buffer (Fig. 5a).

In case of *rac-p-*methylphenylalanine (*rac*-**1c**), the optimal conversion (~50%) and enantiomeric excess (ee_d-**1c**_ > 99%) was obtained after 48 h reaction time using borax and sodium carbonate buffer. Although the reaction proceeded well also in 0.1 M NH_3_ (c = 46%, ee_d-**1c**_** = **96.4%), significantly lower conversion (c = 26%) was obtained when using phosphate-buffer (Fig. 5b).

Probably, the reason for the incomplete reactions obtained using ammonium acetate or 0.1 M NH_3_ buffers (selected for their advantage of easy removal during the reaction work-up), is the occurrence of the reverse ammonia addition reaction, under the low ammonia content. While borax and Tris-buffer systems provide complete conversions for both model substrates, the slight differences in the optimal reaction medium suggest that for rigorous fine-tuning of the efficiency of resolution process, reaction medium tests should be performed for each individual substrate of interest.

#### The effect of biocatalysts: substrate ratio and substrate concentration

Further, the impact of the increased biocatalyst and substrate concentrations on the reactions of model substrates were tested. Firstly, whole-cell *Pc*PAL biocatalysts with various cell densities (OD_600_ of 1, 2, and 4) were used at fixed 2 mM substrate concentration. Generally, the increase of biocatalyst:substrate ratio (2:1 ratio of cell density (OD_600_): mM) resulted shorter reaction times. Thus, when using cell densities of OD_600_ 4, the optimal conversion of ~50% was reached in 5 h in case of *rac*-**1c** and 24 h in case of *rac*-**1k** (Table 2), while lower cell densities of OD_600_ 1 or 2 afforded similar conversions of 50% in longer reaction times (30 h in case of *rac***-1k** and >48 h in case of *rac***-1c**).

**Table 2:** The effect of cell densities upon conversion values in ammonia elimination reactions.

Substrate (2 mM)	Cell density (OD_600_)/biocatalyst: substrate ratio (OD_600_:mM)	c (%)	ee_D-1_ (%)	ee_theorD-1_ (%)
	1/1:2	38.1^a^	63.3	61.7
	2/1:1	39.9^a^	66.3	66.5
	4/2:1	~50^a^	94.1	>99
	1/1:2	27.3^b^	37.5	37.5
	2/1:1	45.6^b^	88.8	84.0
	4/2:1	~50^b^	>99	>99

^a^after 24 h; ^b^after 5 h; ee_theor_ – theoretical enantiomeric excess, calculated by the formula: $\frac{c}{100-c}\times 100$; where c represents the experimentally determined conversion (%); *Reaction conditions*: assays were performed in 1.5 mL polypropylene tubes at 30 °C, 200 rpm, in 500 µL reaction volume, using 2 mM substrate concentration, different ratio of cell density (OD_600_)/substrate concentration (mM) and Tris buffer, pH 8.8 as reaction medium.

The influence of substrate concentration on the resolution process of model substrates was also tested. When increasing the substrate concentration, using the optimal biocatalyst:substrate ratio of 2:1 (OD_600_: mM), the corresponding increase of cell densities was also necessary, leading to the appearance of viscosity issues, hindering reaction monitoring. Therefore, evaluation of the effect of substrate concentrations, ranging between 2 mM and the maximum solubility of model substrates *rac*-**1k** and *rac*-**1c** (70 mM and 15 mM, respectively), was performed with biocatalyst:substrate ratio of 1:2 (OD_600_: mM).

**Figure 6:**
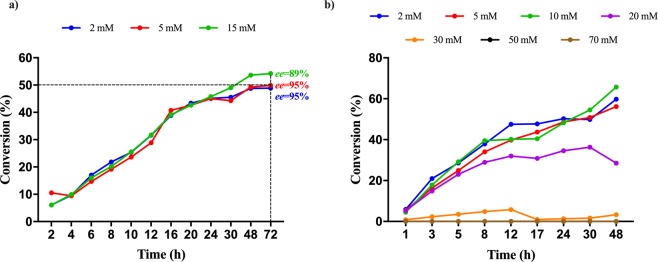
Time-conversion profiles for the ammonia elimination from (**a**) *p*-CH_3_-amino acid *rac*-1c and (**b)** *m*-CF_3_-amino acid *rac*-**1k** using I460V *Pc*PAL, using different substrate concentrations.

In case of ammonia eliminations from *rac*-**1c**, the conversion reached the optimal 50% with similar time progressions at each substrate concentrations tested (Fig. 6a**)**. However, *ee*_D**-1c**_ slightly decreased with the increase of substrate concentration, 95% was obtained at 2 and 5 mM concentration of *rac*-**1c** and conversions approximating the 50% optimal value, while at higher 15 mM substrate concentration ee_D-**1c**_ was 89% (Fig. 6a). Notable, that the obtained *ee* values are slightly lower than the theoretical *ee*s (Figs. S14–S16), supporting a not fully enantioselective resolution process, of which selectivity decreases by the increase of substrate concentration.

**Figure 7:**
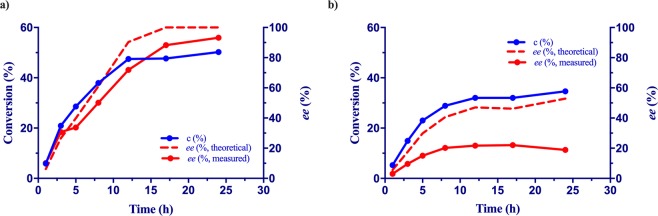
Conversion– and enantiomeric excess–time progression curves for the ammonia eliminations from *m*-CF_3_-amino acid *rac*-**1k** using I460V *Pc*PAL and (**a)** 2 mM and (**b)** 20 mM substrate concentration.

In the ammonia elimination of *rac*-**1k**, using high substrate concentrations, low (c_30mM_ = 6%) or no conversions (at substrate concentration >30 mM) were obtained (Fig. 6b). The decrease of stationary conversion values upon increasing the concentration of *rac*-*m*-(trifluoromethyl)phenylalanine *rac*-**1k** (Fig. 6b), similarly to the ammonia addition onto **2l** (Fig. S11), suggested the occurrence of substrate inhibition. This was confirmed by the kinetic measurements performed using purified *Pc*PAL I460V (Fig. S23). Besides the significant decrease of conversions, at substrate concentrations exceeding 10 mM of *rac*-**1k**, the difference of the *ee* values registered for the unreacted d-**1k** and the theoretical *ee* values also increased, supporting the decrease of the enantioselectivity upon the increase of substrate concentration, similarly as in the case of model substrate *rac*-**1c** (Fig. 7).

Therefore, due to the influence of substrate concentration on the enantioselectivity of the resolution process, and the occurrence of substrate inhibition, the selection of the optimal substrate concentration based primarily on the final ee_d-**1**_ values, followed by the conversion values. Performing the ammonia eliminations of *rac-***1c** and *rac-***1k** under the optimal, relatively low, substrate concentrations (Table 3), high conversions, reaching the theoretical 50% of a kinetic resolution process, and high *ee* values (95% and 93%, respectively) were obtained.

**Table 3:** Conversion and *ee* values of d-1k,c obtained from the corresponding the ammonia elimination reactions performed under optimal conditions^a^.

Substrate	Substituent	*Pc*PAL	[S] (mM)	Time (h)	c (%)	ee_D_ (%)
*rac-***1c**	*p*-CH_3_	I460V	5	48	~50	95
*rac-***1k**	*m*-CF_3_	I460V	2	23	~50	93

^a^*Reaction conditions*: assays were performed in 1.5 mL polypropylene tubes at 30 °C, 200 rpm for 16 h, in 500 µL reaction volume, using the corresponding substrate concentration, ratio of cell density (OD_600_)/substrate concentration (mM) of 1.0, and Borax buffer as reaction medium.

### Preparative scale biotransformations

**Figure 8:**
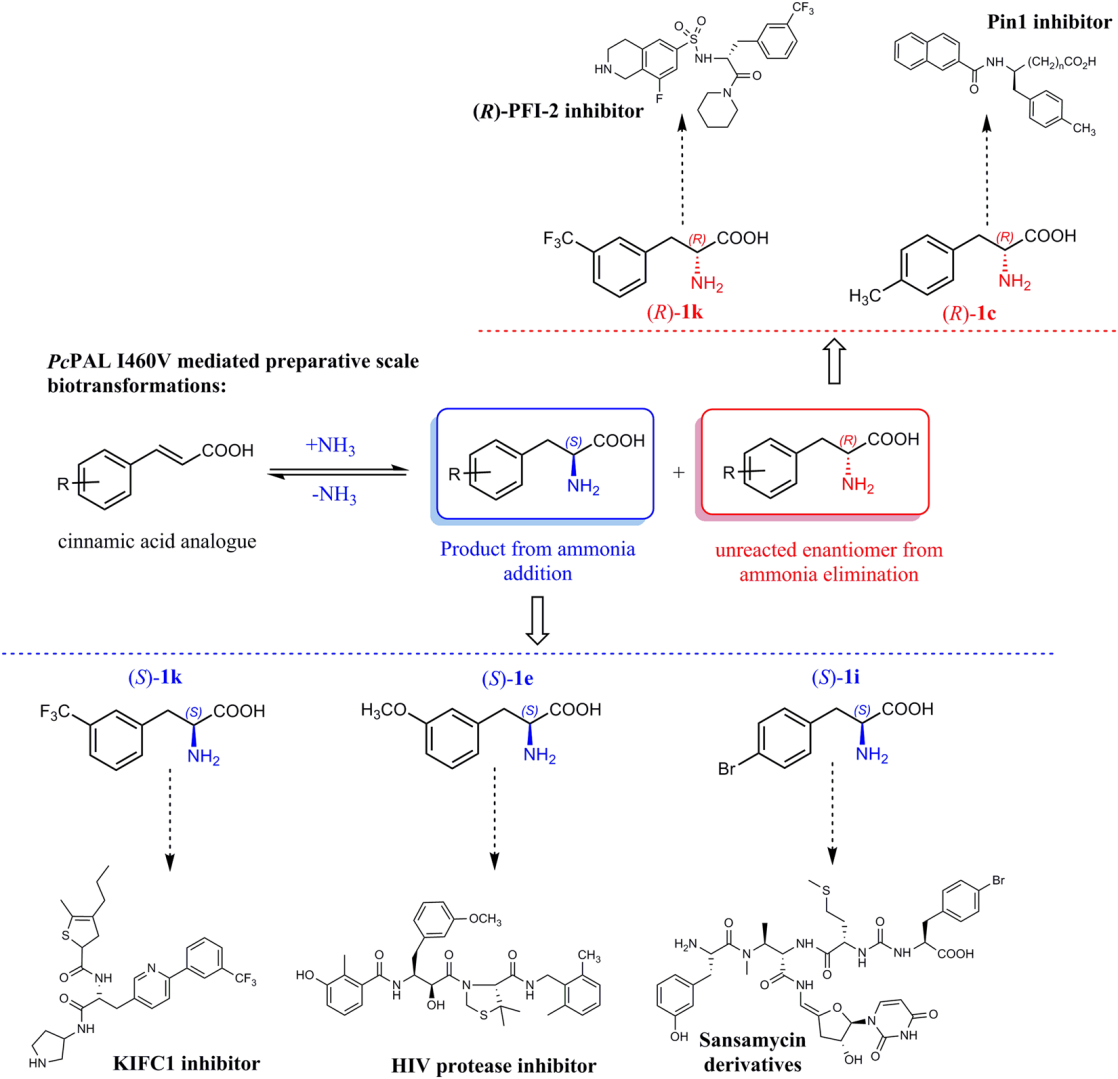
Preparative scale ammonia additions and ammonia eliminations providing access to synthetically valuable phenylalanine analogues.

Accordingly, to obtain synthetically important Phe analogues in high optical purity, the optimized ammonia additions onto substrates **2e, 2i** and **2k** and ammonia eliminations from *rac***-1c**, *rac***-1k** were performed at preparative scale (500 mg) (Fig. 8).

Using the optimal reaction conditions set-up by analytical scale biotransformations, the preparative scale biotransformation provided similarly high conversion and *ee* values, affording the corresponding d- and l- amino acids in good conversions, isolated yields and optical purities (Table 4). The efficiency of ammonia eliminations, in terms of yields and enantiomeric excess values, are comparable with the kinetic resolutions performed with purified/commercial enzymes, such as the penicillin G acylase mediated enantioselective acylation for d-*p*-methylphenylalanine d-**1c**^44^, or the l-AAO/catalase catalyzed kinetic resolution process yielding d-*m*-(trifluoromethyl)phenylalanine d-**1k**^45^, a key chiral intermediate for (*R*)-PFI-2^33^. Definitely, in terms of conversions the kinetic resolution-type ammonia elimination of *rac*-**1c** is surpassed by the asymmetric synthetic routes, such as the reductive amination of the corresponding keto-acid catalyzed by DAADH (d-amino acid dehydrogenase)^46^, necessitating co-factor (NADPH) regeneration system or the l-amino acid deaminase and engineered aminotransferase coupled cascade, that uses l- or racemic amino acid **1c** as starting material^47^. In this context the asymmetric synthesis-type ammonia additions, starting from inexpensive cinnamic acids, represent a more attractive synthetic route. Accordingly, in case of l-*m*-(trifluoromethyl)phenylalanine l-**1k**, intermediate for kinesin KIFC1 inhibitors^32^, the developed PAL-ammonia addition provides higher yield than the claimed enantioselective hydrolysis of corresponding carboxylic esters^48^. The yields for l-*m*-methoxyphenylalanine l-**1e** are comparable with the yields of the recently reported, *Pb*PAL mediated semi-preparative scale reaction (61% yield, ee > 99%)^40^, but also with the aspartate aminotransferase catalyzed reductive amination^49^. The optically pure l-*p*-bromophenylalanine l-**1i**, as versatile chiral intermediate^12^ was also obtained in high, 82% yield (Table 4), similarly as in case of using improved *Av*PAL variants^12^, but somewhat lower than the 94% reported yield when using the 31E variant of *Rhodotorula graminis* PAL *(RgrPAL)*^24^.

**Table 4:** Preparative scale ammonia addition and ammonia elimination reactions.

	Product	Reaction time (h)	Y^b^ (%)	ee (%)	${[{\boldsymbol{\alpha }}]}_{{\boldsymbol{D}}}^{{\bf{27}}}$^c^
Ammonia addition	(*S*)-2-amino-3-(3-methoxyphenyl)propanoic acid, (*S*)**-1e**	42	59	>99	−66.4
	(*S*)-2-amino-3-(4-bromophenyl)propanoic acid, (*S*)**-1i**	48	82	>99	−47.6
	(*S*)-2-amino-3-(3-(trifluoromethyl)phenyl)propanoic acid, (*S*)**-1k**	48	52	>99	−35.2
Ammonia elimination^*a*^	(*R*)-2-amino-3-(*p*-tolyl)propanoic acid, (*R*)**-1c**	30	49	95	+5.7
	(*R*)-2-amino-3-(3-(trifluoromethyl)phenyl)propanoic acid, (*R*)**-1k**	30	39	93	+31.5

^a^At ~50% conversion; ^b^The reaction yields were determined from the preparative scale reaction including product isolation, purification steps (for reaction conditions see ESI, chapter **4**); ^c^The measurements were performed: in MeOH with substrate concentration of 10 mg/1 mL.

## Methods

### Preparation of whole cell biocatalysts for enzymatic reactions - general procedure

The overnight preculture was prepared in Erlenmeyer flasks containing LB (Luria Bertani) medium supplemented with carbenicillin (50 µg/mL) and chloramphenicol (30 µg/mL) and inoculated with glycerol stocks of *E. coli* Rosetta (DE3) pLysS cells harbouring the pET19b vector carrying the *wt*- or mutant *pcpal* gene^50^, followed by overnight incubation at 37 °C and shaking at 200 rpm. The obtained preculture (5 mL) was further used to inoculate shake flasks (2l) containing 500 mL LB. Cultures were grown at 37 °C, 200 rpm until OD_600_ reached 0.6–0.8, at which point protein production was induced via the addition of 0.1 mM IPTG (final concentration), and the cell growth was maintained at 25 °C for another 16 h. Cell densities of OD_600_ were measured after for each mutant variant and wild-type *Pc*PAL. The cells were harvested by centrifugation at 4000 rpm (1751 × g) and 4 °C for 20 min and washed with PBS buffer (20 mM phosphate, 150 mM NaCl, pH 8.0) (4000 rpm, 1751 × g, 4 °C, 20 min) and stored at -20 °C in 1.5 ml polypropylene tubes with OD_600_ of ~2 until further use.

### Biotransformation screenings for ammonia addition reactions - general procedure

The ammonia addition reactions were performed in duplicate using 1.5 ml polypropylene tubes, containing the whole cell *Pc*PAL-biocatalysts, prepared as described above. The bacterial pellet was resuspended to an OD_600_ of ~2, in 1 mL of ammonia source (2, 4, 6 M NH_4_OH pH 10 adjusted with CO_2_) and ammonium carbamate (2, 4, 6 M NH_4_[H_2_NCO_2_], pH 9.6–10 without adjustment). For the biotransformations, 2 mM substrate (cinnamic acids 2a-l) concentrations were used, and the reaction mixtures were incubated at 30 °C, 250 rpm for specified reaction times. Conversions were monitored using reversed-phase high performance liquid chromatography (HPLC). Reaction samples were quenched by adding an equal volume of MeOH, vortexed and centrifuged (13400 rpm, 12000 × g, 10 min). The supernatant was filtered through a 0.22 μm nylon membrane filter and analyzed by HPLC.

### Biotransformation screenings for ammonia elimination reactions- general procedure

The ammonia elimination reactions were performed in duplicate using 1.5 ml polypropylene tubes, containing the whole cell *Pc*PAL -biocatalysts, prepared as described above. The bacterial pellet was resuspended to an OD_600_ of ~2, in 1 mL of different buffers: Tris (20 mM Tris.HCl, 120 mM NaCl, pH 8.8,), NH_3_-buffer (0.1 M NH_4_OH, pH 9.5, adjusted with CO_2_), borax (0.1 M Na_2_[B_4_O_5_(OH)_4_], pH 9.5), ammonium acetate buffer (0.1 M, pH 9.5,) sodium carbonate (0.1 M, pH 9.0) and phosphate-buffer (0.1 M phosphate, pH 8.8). For the biotransformations 2 mM substrate (racemic amino acids *rac-*1a-l) concentrations were used, and the reaction mixtures were incubated at 30 °C, 250 rpm for different reaction times. Conversions were monitored using reversed-phase HPLC. Reaction samples were quenched by adding an equal volume of MeOH, vortexed and centrifuged (13400 rpm, 12000 × g, 10 min). The supernatant was filtered through a 0.22 μm nylon membrane filter and analyzed by HPLC.

### Preparative scale ammonia additions

In a 500 mL flask, *E. coli* Rosetta (DE3) pLysS cells harbouring the pET19b vector carrying the corresponding mutant *pcpal* gene were resuspended in 6 M NH_4_OH-solution (pH 9.8 adjusted with CO_2_) to give a final OD_600_ of ~10 for 2i and 2k (13.26 g wet cells in 221 mL reaction volume and 13.92 g wet cells in 232 mL reaction volume, respectively) and a final OD_600_ of ~30 (16.92 g wet cells in 94 mL reaction volume) for 2e. 0.5 g cinnamic acid 2e, 2i, 2k (2.8, 2.2, 2.3 mmol, respectively) was added to the cell suspension in a final concentration of 30 mM for 2e and 10 mM for 2i, 2k and the reaction was incubated at 200 rpm, 30 °C for 48 hours, monitoring the conversion values by reversed-phase HPLC. When stationary conversions were reached (Figs. 4a,b and S9) the reaction mixture was acidified to pH 1.5 by dropwise addition of aqueous H_2_SO_4_ (50% w/v). The formed precipitate was removed by centrifugation at 10000 rpm (10947 × g), 4 °C for 20 min., while the non-reacted cinnamic acid was removed by extraction with ethyl acetate (3 × 10 mL). The amino acid found within the aqueous phase was purified by ion exchange chromatography using Dowex® 50WX2 resin, using 2 M NH_4_OH solution for elution. The fraction containing the final product was evaporated in a centrifugal evaporator affording the pure l-amino acid (0.29 g, 1.63 mmol, 59% isolation yield for (*S*)-1e; 0.4 g, 1.76 mmol, 80% isolation yield for (*S*)-1i and 0.26 g, 1.2 mmol, 52% isolation yield for (*S*)-1k).

### Preparative scale ammonia eliminations

In a 500 mL flask, *E. coli* Rosetta (DE3) pLysS cells harbouring the pET19b vector carrying the corresponding or mutant *pcpal* gene were resuspended in Borax buffer (pH 10 unadjusted) to give a final OD_600_ of ~7.5 (12.51 g wet cell mass /278 mL for *rac*-1c and 6.43 g wet cell mass /143 mL *rac*-1k). Racemic phenylalanine analogue *rac*-1c or *rac*-1k (0.25 g, 1.4 mmol, or 0.5 g, 2.1 mmol, respectively) was added to the cell suspension in a final concentration of 5 or 15 mM, respectively and the reactions were incubated at 200 rpm, 30 °C for 48 hours, monitoring the conversion values by reversed-phase HPLC (Figs. S11, S12). When stationary conversions were reached, the reaction mixtures were purified using the method described above for the preparative scale ammonia addition, providing the d- amino acids (0.245 g, 1.34 mmol 49% isolation yield for (*R*)-1c and 0.19 g, 0.82 mmol and 39% isolation yield for (*R*)-1k).

## Conclusions

Within this study, we developed highly efficient biocatalytic procedures for the synthesis of valuable enantiopure l- and d- Phe, using as whole-cell biocatalyst recombinant *E. coli* cells harbouring the plasmid of *Pc*PAL mutants (L256V, L134A, I460V), specifically tailored towards the targeted ring-substituted cinnamic acid and phenylalanine substrates.

In case of ammonia addition reactions, regarding to substrate solubility and reaction time, the optimal reaction medium was found to be 6 M NH_4_OH, while in several cases the substrate concentration significantly affected the final conversions. Substrates bearing *ortho*-substituents or *para*-electron donating substituents were transformed with high conversions (66–91%) even at high substrate concentrations (30–70 mM), while in case of substrates with *meta*- or electron withdrawing *para-*substituents high substrate concentrations (>2–10 mM) significantly lowered the final conversion values, substrate inhibition occurring in several cases.

In case of ammonia elimination reactions, the reaction medium affected slightly, but differently the kinetic resolution of the model substrates, suggesting specific conditions for each substrate of interest. Using an optimal biocatalyst:substrate ratio, inhibitory effect occurred at high substrate concentration (>2–5 mM), while the enantioselectivity of the resolution process also decreased, forcing the use of relatively low substrate concentrations.

Finally, the preparative scale ammonia addition reactions of *m*-methoxy-cinnamic acid **2e**, *p*-bromo-cinnamic acid **2i**, *m*-(trifluoromethyl)cinnamic acid **2k** and ammonia elimination reactions of *rac*-*p*-methylphenylalanine *rac*-**1c**, *rac*-*m*-(trifluoromethyl)phenylalanine *rac*-**1k** were performed under their specific optimal conditions, producing in high yields and *ee*s the corresponding, highly valuable l- and d- phenylalanines.

## Supplementary information

Supplementary Information

## References

[1] Wakiec R (2008). Enhanced susceptibility to antifungal oligopeptides in yeast strains overexpressing ABC multidrug efflux pumps. Antimicrob. Agents Chemother..

[2] Slaninová J, Maletínská L, Vondrášek J, Procházka Z (2001). Magnesium and biological activity of oxytocin analogues modified on aromatic ring of amino acid in position 2. J. Peptide Sci..

[3] Parmeggiani F, Weise NJ, Ahmed ST, Turner NJ (2018). Synthetic and therapeutic applications of ammonia-lyases and aminomutases. Chem. Rev..

[4] Heberling MM, Wu B, Bartsch S, Janssen DB (2013). Priming ammonia lyases and aminomutases for industrial and therapeutic applications. Curr. Opin. Chem. Biol..

[5] Liu W (1999). (Great Lakes Chemical Co.), US Pat 5,981,239, 1999. [Chem. Abstr..

[6] Turner N (2011). Ammonia lyases and aminomutases as biocatalysts for the synthesis of α-amino and β-amino acids. Curr. Opin. Chem. Biol..

[7] Dreßena A, Hilberatha T, Mackfeld U, Rudatb J, Pohla M (2017). Phenylalanine ammonia lyase from *Arabidopsis thaliana* (*At*PAL2): A potent MIO-enzyme for the synthesis of non-canonical aromatic alpha-amino acids. Part II: Application in different reactor concepts for the production of (*S*)-2-chloro-phenylalanine. J. Biotechnol..

[8] Nagy EZA (2019). Mapping the hydrophobic substrate binding site of phenylalanine ammonia lyase from *Petroselinum crispum*. ACS Catal..

[9] Filip A (2018). Tailored mutants of phenylalanine ammonia-lyase from *Petroselinum crispum* for the synthesis of bulky l- and d-arylalanines. ChemCatChem..

[10] Lovelock SL, Turner NJ (2014). Bacterial *Anabaena variabilis* phenylalanine ammonia lyase: A biocatalyst with broad substrate specificity. Bioorg. Med. Chem..

[11] Weise NJ (2016). Intensified biocatalytic production of enantiomerically pure halophenylalanines from acrylic acids using ammonium carbamate as the ammonia source. Catal. Sci. Technol..

[12] Ahmed ST, Parmeggiani F, Weise NJ, Flitsch SL, Turner NJ (2015). Chemoenzymatic Synthesis of optically pure L- and D-biarylalanines through biocatalytic asymmetric amination and palladium-catalyzed arylation. ACS Catal..

[13] Jia SR, Cui JD, Yan L, Sun AY (2008). Production of L-phenylalanine from trans-cinnamic acids by high-level expression of phenylalanine ammonia lyase gene from *Rhodosporidium toruloides* in *Escherichia coli*. Biochem. Eng. J..

[14] Cui JD, Qiu JQ, Fan XW, Jia SR, Tan ZL (2014). Biotechnological production and applications of microbial phenylalanine ammonia lyase: a recent review. Crit. Rev. Biotechnol..

[15] de Lange B (2011). Asymmetric synthesis of (*S*)-2-indolinecarboxylic acid by combining biocatalysis and homogeneous catalysis. ChemCatChem..

[16] Gloge A, Zoń J, Kővári. Á, Poppe L, Rétey J (2000). Phenylalanine ammonia-lyase: The use of its broad substrate specificity for mechanistic investigations and biocatalysis - Synthesis of L-arylalanines. Chem. Eur. J..

[17] Paizs C, Katona A, Rétey J (2006). The interaction of heteroarylacrylates and alanines with phenylalanine ammonia-lyase from parsley. Chem. Eur. J..

[18] Paizs C (2010). 2-amino-3-(5-phenylfuran-2-yl)propionic acids and 5-phenylfuran-2-ylacrylic acids are novel substrates of phenylalanine ammonia-lyase. Heterocycles..

[19] Bartha-Vári JH (2015). Immobilization of phenylalanine ammonia lyase on single-walled carbon nanotubes for stereoselective biotransformations in batch and continuous-flow modes. ChemCatChem..

[20] Bartha-Vári JH (2017). Aminated single-walled carbon nanotubes as carrier for covalent immobilization of phenylalanine ammonia-lyase. Period. Polytech. Chem. Eng..

[21] Baedeker M, Schulz GE (1999). Overexpression of a designed 2.2 kb gene of eukaryotic phenylalanine ammonia-lyase in *Escherichia coli*. FEBS Lett..

[22] Bencze LC (2017). Expanding the substrate scope of phenylalanine ammonia-lyase from *Petroselinum crispum* towards styrylalanines. Org. Biomol. Chem..

[23] Bartsch S, Bornscheuer UT (2010). Mutational analysis of phenylalanine ammonia lyase to improve reactions rates for various substrates. Protein Eng. Des. Sel..

[24] Rowles I (2016). Engineering of phenylalanine ammonia lyase from *Rhodotorula graminis* for the enhanced synthesis of unnatural l-amino acids. Tetrahedron.

[25] Ahmed ST, Parmeggiani F, Weise NJ, Flitsch SL, Turner NJ (2018). Engineered ammonia lyases for the production of challenging electron-rich l-phenylalanines. ACS Catal..

[26] Wu B, Szymanski W, Heberling MM, Feringa BL, Janssen DB (2011). Aminomutases: mechanistic diversity, biotechnological applications and future perspectives. Trends. Biotech..

[27] Wu B (2012). Mechanism-inspired engineering of phenylalanine aminomutase for enhanced β-regioselective asymmetric amination of cinnamates. Angew. Chem..

[28] Bartsch S (2013). Redesign of a phenylalanine aminomutase into a phenylalanine ammonia lyase. ChemCatChem.

[29] Wu B (2012). Mechanism-inspired engineering of phenylalanine aminomutase for enhanced β-regioselective asymmetric amination of cinnamates. Angew. Chem. Int. Ed..

[30] Wu B (2009). Enzymatic synthesis of enantiopure alpha- and beta-amino acids by phenylalanine aminomutase-catalysed amination of cinnamic acid derivatives. ChemBioChem.

[31] Szymanski W (2009). Phenylalanine aminomutase-catalyzed addition of ammonia to substituted cinnamic acids: a route to enantiopure α- and β-amino acids. J. Org. Chem..

[32] Yang B (2014). Discovery of potent KIFC1 inhibitors using a method of integrated high-throughput synthesis and screening. J. Med. Chem..

[33] Lenstra DC (2018). Structure-activity relationship studies on (*R*)-PFI-2 analogs as inhibitors of histone lysine methyltransferase SETD7. ChemMedChem..

[34] Dong L (2010). Structure-based design of novel human Pin1 inhibitors (II). Bioorg. Med. Chem. Lett..

[35] Hwang TL (2013). Design and synthesis of tryptophan containing dipeptide derivatives as formyl peptide receptor 1 antagonist. Org. Biomol. Chem..

[36] Arava VR (2013). Asymmetric synthesis of unnatural amino acids and Tamsulosin chiral intermediate. Synth. Commun..

[37] Mimoto T (2004). Structure–activity and structure–metabolism relationships of HIV protease inhibitors containing the 3-hydroxy-2-methylbenzoylallophenylnorstatine structure. Bioorg. Med. Chem..

[38] Dreßena A (2017). Phenylalanine ammonia lyase from *Arabidopsis thaliana* (*At*PAL2): A potent MIO-enzyme for the synthesis of non-canonical aromatic alpha-amino acids Part I: Comparative characterization to the enzymes from *Petroselinum crispum* (*Pc*PAL1) and *Rhodosporidium toruloides* (*Rt*PAL). J. Biotechnol..

[39] Parmeggiani F, Lovelock SL, Weise NJ, Ahmed ST, Turner NJ (2015). Synthesis of d- and l-phenylalanine derivatives by phenylalanine ammonia lyases: A multienzymatic cascade process. Angew. Chem. Int. Ed..

[40] Weise NJ (2017). Zymophore identification enables the discovery of novel phenylalanine ammonia lyase enzymes. Sci. Rep..

[41] Hyun MW, Yun YH, Kim JY, Kim SH (2011). Fungal and plant phenylalanine ammonia-lyase. Mycobiology.

[42] Michelle CM (2007). Discovery of two cyanobacterial phenylalanine ammonia lyases: kinetic and structural characterization. Biochemistry..

[43] Fan Z (2017). Modulating the pH activity profiles of phenylalanine ammonia lyase from *Anabaena variabilis* by modification of center-near surface residues. Appl. Biochem. Biotechnol..

[44] Gong X, Su E, Wang P, Wei D (2011). *Alcaligenes faecalis* penicillin G acylase-catalyzed enantioselective acylation of dl-phenylalanine and derivatives in aqueous medium. Tetrahedron Lett..

[45] Pirrung MC, Krishnamurthy N (1993). Preparation of (*R*)-phenylalanine analogues by enantioselective destruction using l-amino acid oxidase. J. Org. Chem..

[46] Parmeggiani F (2016). Single-biocatalyst synthesis of enantiopure d-arylalanines exploiting an engineered d-amino acid dehydrogenase. Adv. Synth. Catal..

[47] Walton CJW (2018). Engineered aminotransferase for the production of D-phenylalanine derivatives using biocatalytic cascades. ChemCatChem.

[48] Hans Ullrich, H., Merten, S. & Reinhold, K. (From Ger. Offen.) DE 3733506 A1 19890413 (1989).

[49] Yu J (2019). Chemoenzymatic synthesis of l-3,4-dimethoxyphenyl-alanine and its analogues using aspartate aminotransferase as a key catalyst. Catal. Commun..

[50] Dima NA (2016). Expression and purification of recombinant phenylalanine ammonia-lyase from Petroselinum crispum. Stud. Univ. Babes-Bolyai Ser. Chem..

