# Supplementary material for: The production of l- and d-phenylalanines using engineered phenylalanine ammonia lyases from Petroselinum crispum
Source: Sci Rep. 2019 Dec 27;9:20123. doi: 10.1038/s41598-019-56554-0 (PMC6934771; doi:10.1038/s41598-019-56554-0)
Supplement: Supplementary file 1 — Supplementary Information [file 41598_2019_56554_MOESM1_ESM.pdf]

Supporting Information

The production of L- and D-phenylalanines using engineered phenylalanine ammonia lyases from *Petroselinum crispum*

Souad Diana Tork[1]†, Emma Zsófia Aletta Nagy[1]†, Lilla Cserepes[1], Diana Monica Bordea[1], Botond Nagy[1], Monica Ioana Toşa[1], Csaba Paizs[1], László Csaba Bencze[1]*

[1] Biocatalysis and Biotransformation Research Center, Faculty of Chemistry and Chemical Engineering, Babeș-Bolyai, University of Cluj-Napoca, Arany János Str. 11, RO-400028 Cluj-Napoca, Romania

***** Correspondence: cslbencze@chem.ubbcluj.ro

†these authors contributed equally to this work

Table of Contents

1. Materials **3**

2. Instrumentation **3**

3. Experimental Section **4**

3.1. Optimization procedures **4**

3.2 pH profile of biotransformation with whole cells of *Pc*PAL **5**

3.3. Substrate concentration optimizations **5**

3.4. HPLC chromatograms from the preparative scale biotransformations **13**

3.5. Substrate inhibition in the ammonia addition reaction of *p*-CF_3-cinnamic acid 2l catalysed by I460V *Pc*PAL **15**

3.5. Substrate inhibition in the ammonia elimination reaction of *m*-CF_3-amino acid *rac*-1k catalysed by I460V *Pc*PAL **16**

3.6. Product inhibition in the ammonia addition reaction of *p*-CH_3O-cinnamic acid 2f catalysed by I460V *Pc*PAL **16**

4. References **18**

1. Materials

The commercial chemicals and solvents were products of Sigma Aldrich and Alfa-Aesar. IPTG was purchased from Thermo Fischer Scientific (Waltham, MA, USA), LB medium from Liofilchem (Roseto, Italy), while protease inhibitor cocktail was obtained from Hoffman La-Roche (Basel, Switzerland). The synthesis of substrates *rac*-**1a-l** and **2a-l** was performed accordingly to procedures from our earlier works[1]. The *Pc*PAL mutants used within this study were obtained in our previous works[1,2]. The isolation and purification of mutant I460V and L134A *Pc*PAL was performed using a reported protocol[3].

2. Instrumentation

The ^{1}H and ^{13}C NMR spectra were recorded on Bruker (Billerica, MA, USA) Advance spectrometers operating at 400 MHz and 101 MHz / 600 MHz and 151 MHz, respectively. Spectra were recorded at 25 °C in $CDCl_3$, D_2O, MeOD-d_4, DMSO-d_6. ^{1}H and ^{13}C NMR spectra were referenced internally to the solvent signal. MS spectra and LC-MS analysis were recorded on Agilent 6410 Triple Quadrupole LC-MS/MS mass spectrometry system. LC-MS measurements were performed using Phenomenex Kynetex 2,6μm C18, 100 Å, 50×21mm column, acetonitrile- water (0.1% HCOOH) 70:30 (V/V) as mobile phase at 0.3 mL/min flow rate. The MS detector was operated in positive/negative electrospray ionization mode, with source temperature of 350 ^{o}C, capillary voltage 4000V, fragmentor 120V and in Scan mode, at least +/- 50 amu around molecular ion. High performance liquid chromatography (HPLC) analyses were conducted with an Agilent (Santa Clara, CA, USA) 1200, 1260 and 1100 systems. Thin layer chromatography (TLC) was carried out using Merck Kieselgel 60F254 sheets. Spots were visualized by treatment with 5% ethanolic phosphomolybdic acid or ninhydrin solution and heating. Preparative chromatographic separations were performed using column chromatography on Merck Kieselgel 60 (63-200 μm). Optical rotations were determined on Autopol® IV Automatic polarimeter. The HPLC monitoring of the enzymatic reactions, the determination of conversion and enantiomeric excess values by HPLC was performed using the previously developed analytical methods[1].

3. Experimental Section

3.1. Optimization procedures

Table S1. The optimized parameters, the reaction condition of the corresponding optimization step and the resulted optimal parameters in case of ammonia addition reactions

Parameter	Conditions	Optimal parameter
biocatalysts: substrate ratio	Whole cells of OD_{600} of ~ 1, 2, 4, 8 (corresponding to ~ 6, 12, 24, 48 mg/mL wet cell concentration) in 200 µL reaction volume for 2 mM substrate concentration	OD_{600} ~2 (~12 mg /mL wet cell concentration)
ammonia concentration	2 mM substrate concentration in ammonia (2, 4, 6 M NH_4OH pH 10 adjusted with CO_2) and ammonium carbamate (2, 4, 6 M, pH 9.6 - 10 without adjustment), using 1:1 biocatalysts: substrate ratio	6 M NH_4OH
substrate concentration	500 µL reaction volume, 2-70 mM substrate concentration, ratio of cell density (OD_{600}) / substrate concentration (mM) of 1.0; 6 NH_4OH at pH 10 (adjusted with CO_2) reaction medium; assays were performed in 1.5 mL polypropylene vials at 30 °C, 200 rpm for 16 h	Specific for each substrate (see Chapter 3.3 in ESI)

Table S2. The optimized parameters, reaction conditions of the corresponding optimization step and the resulted optimal parameters in case of ammonia elimination reactions

Parameter	Conditions	Optimal parameter
biocatalysts: substrate ratio	Whole cells of OD_{600} of ~ 1, 2, 4 (corresponding to ~ 6, 12, 24 mg/mL wet cell concentration) in 200 µL reaction volume for 2 mM substrate concentration	OD_{600} ~1 (~6 mg/mL wet cell concentration)
pH optimization	2 mM substrate concentration in Tris-buffers of pH values 7.5, 7.8, 8.1, 8.6, 8.8, 9.0, 9.4, 9.6, 9.9 using whole cells biocatalyst of OD_{600} ~1	pH 8.8-9.5 (see Chapter 3.2 in ESI)
Reaction medium	2 mM substrate concentration Tris (20 mM Tris, 120 mM NaCl, pH 8.8,), NH_3-buffer (0.1 M NH_4OH, pH 9.5, adjusted with CO_2), 10 mM borax (0.1 M, pH 9.5), ammonium acetate buffer (0.1 M, pH 9.5) sodium carbonate (0.1 M, pH 9.0) and phosphate-buffer (0.1 M phosphate, pH 8.8) using biocatalysts substrate ratio of 1:2	10 mM borax (0.1 M, pH 9.5)
substrate concentration	500 µL reaction volume, 2 mM substrate concentration, different ratio of cell density (OD_{600}) / substrate concentration (mM); Tris buffer pH 8.8 as reaction medium.	Specific for each substrate (see Chapter 3.3 in ESI)

3.2 pH profile of biotransformation with whole cells of *Pc*PAL

The conversion of the I460V *Pc*PAL-whole cells mediated ammonia elimination of model substrate *rac*-**1k**, using as reaction medium different Tris-buffers with pH value ranging between pH 7.5–10.0 was monitored by HPLC (**Figure S1**). The reactions were performed at 30 ˚C, 200 rpm, for 20 h reaction time.

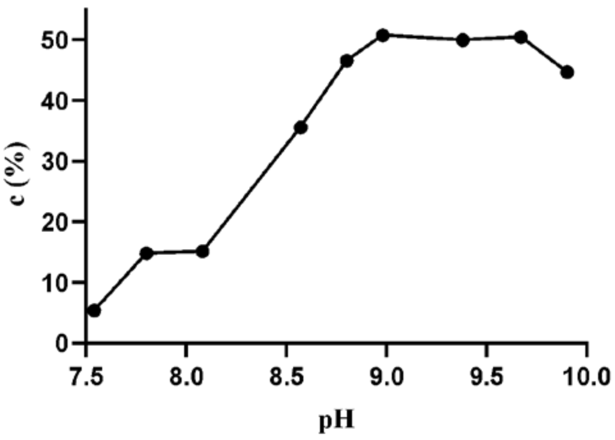

Figure S1. Conversion of *rac*-**1k** using *Pc*PAL I460V whole cells biocatalyst in Tris-buffer with pH value ranging between pH 7.5–9.9 (conversion values obtained after 20 h reaction times are represented)

3.3. Substrate concentration optimizations

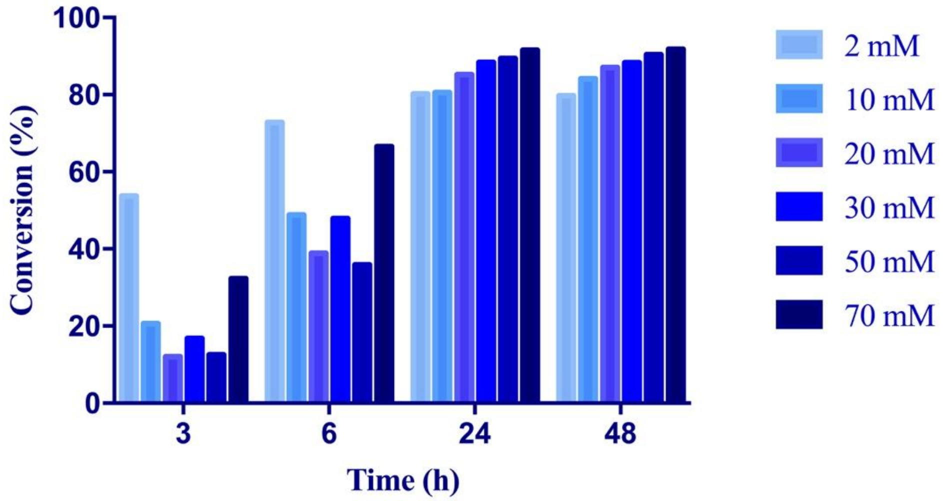

Figure S2. Time conversion profile for the ammonia addition onto *o*-CH_3-cinnamic acid **2a** using L256V *Pc*PAL.

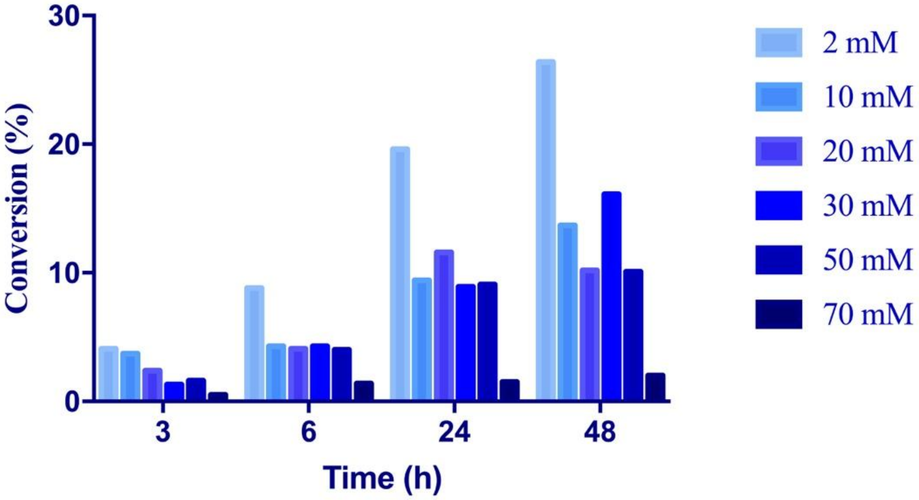

Figure S3. Time conversion profile for the ammonia addition onto *m*-CH_3-cinnamic acid **2b** using L134A *Pc*PAL.

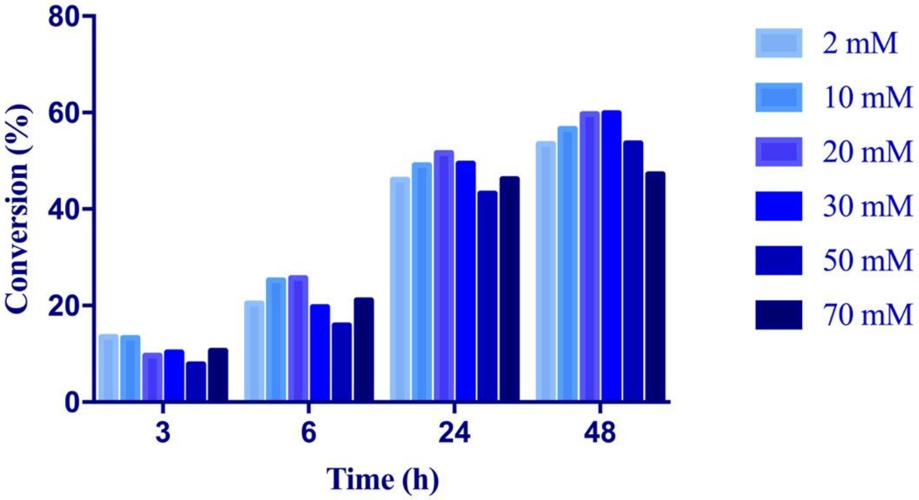

Figure S4. Time conversion profile for the ammonia addition onto *p*-CH_3-cinnamic acid **2c** using I460V *Pc*PAL.

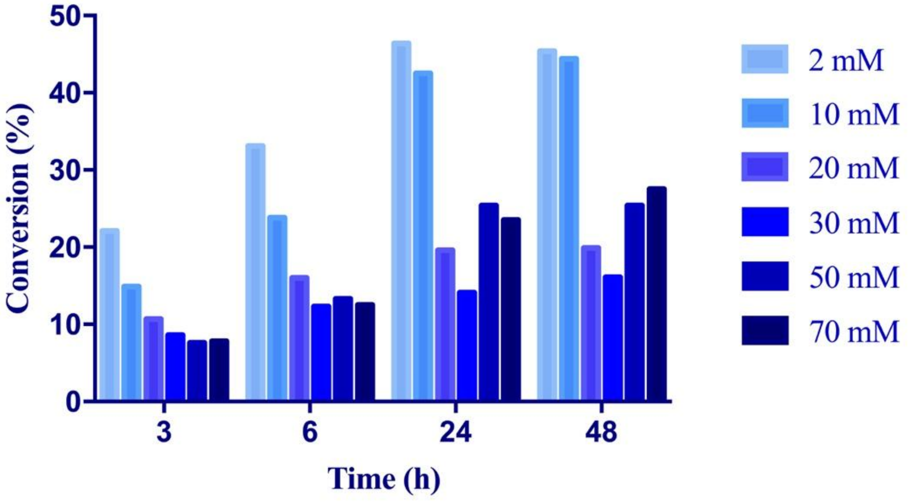

Figure S5. Time conversion profile for the ammonia addition onto *o*-OCH_3-cinnamic acid **2d** using L134A *Pc*PAL.

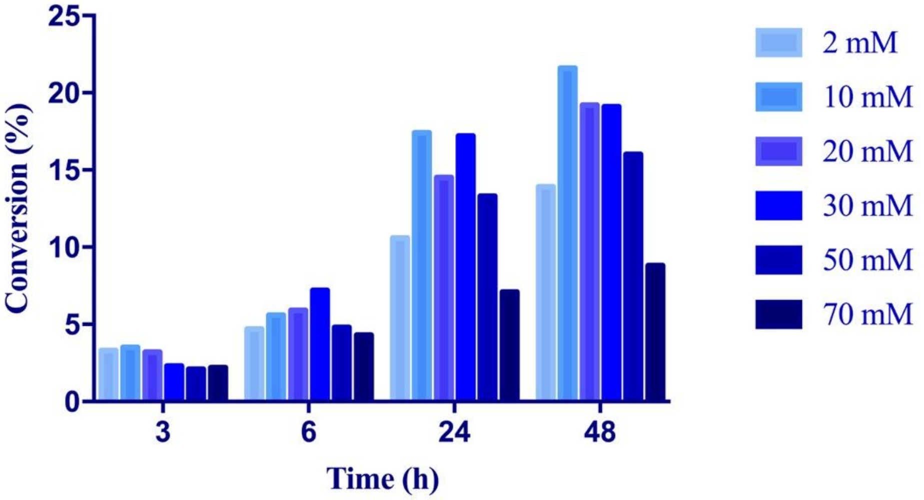

Figure S6. Time conversion profile for the ammonia addition onto *p*-OCH_3-cinnamic acid **2f** using I460V *Pc*PAL.

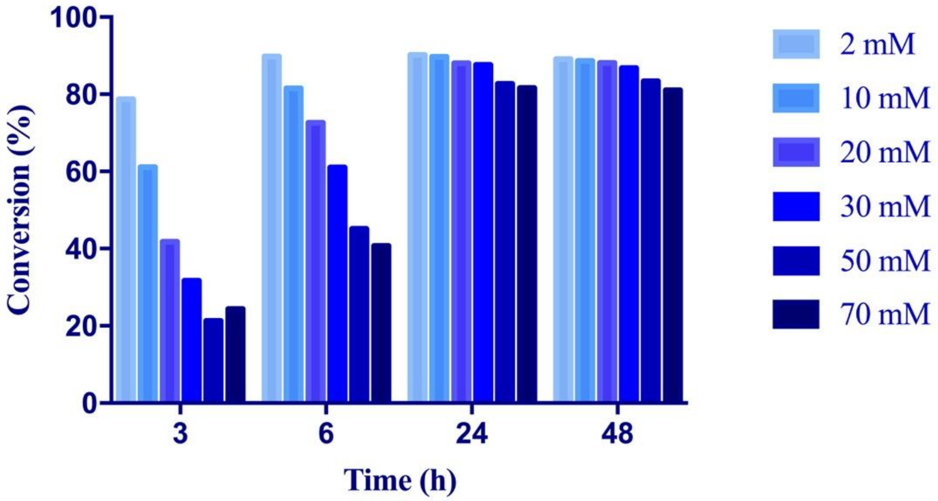

Figure S7. Time conversion profile for the ammonia addition onto *o*-Br-cinnamic acid **2g** using L256V *Pc*PAL.

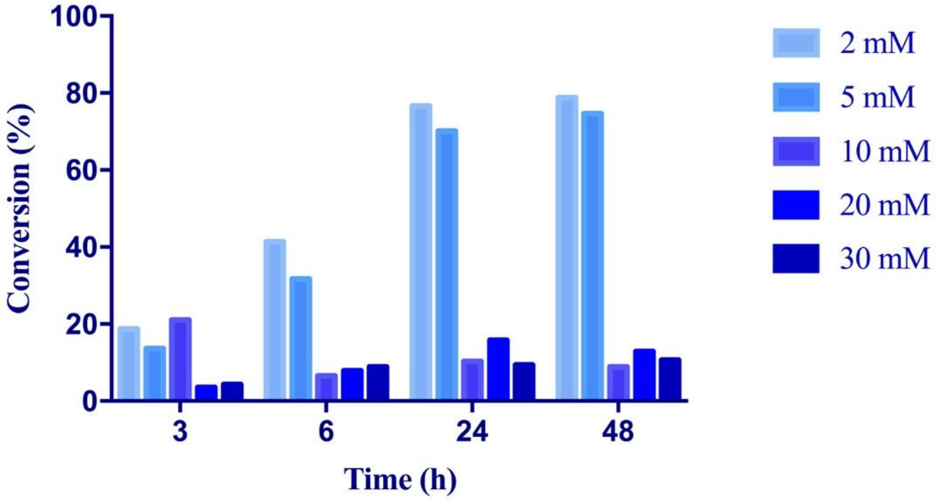

Figure S8. Time conversion profile for the ammonia addition onto *m*-Br-cinnamic acid **2h** using I460V *Pc*PAL.

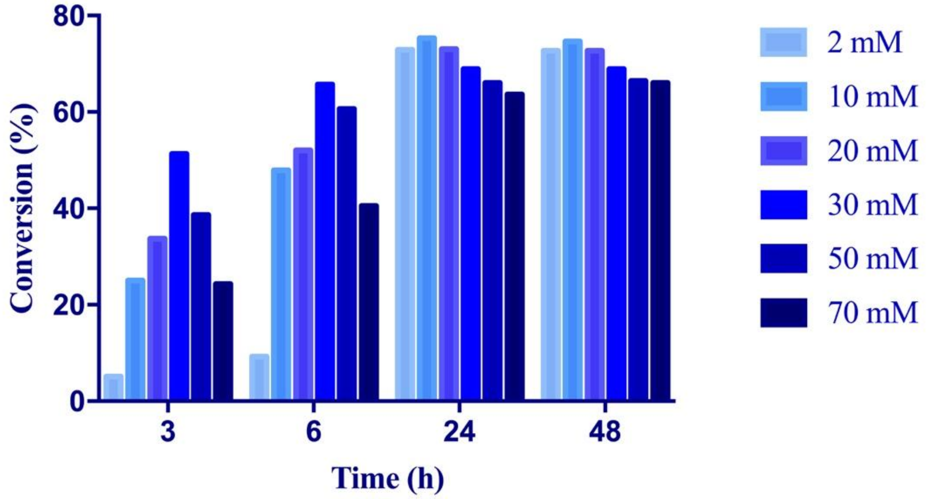

Figure S9. Time conversion profile for the ammonia addition onto *o*-CF_3-cinnamic acid **2j** using L256V *Pc*PAL.

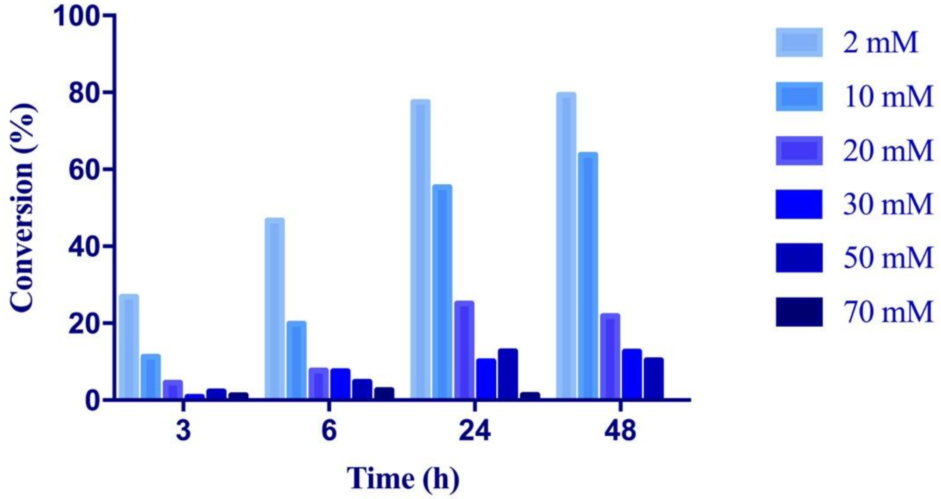

Figure S10. Time conversion profile for the ammonia addition onto *m*-CF_3-cinnamic acid **2k** using I460V *Pc*PAL.

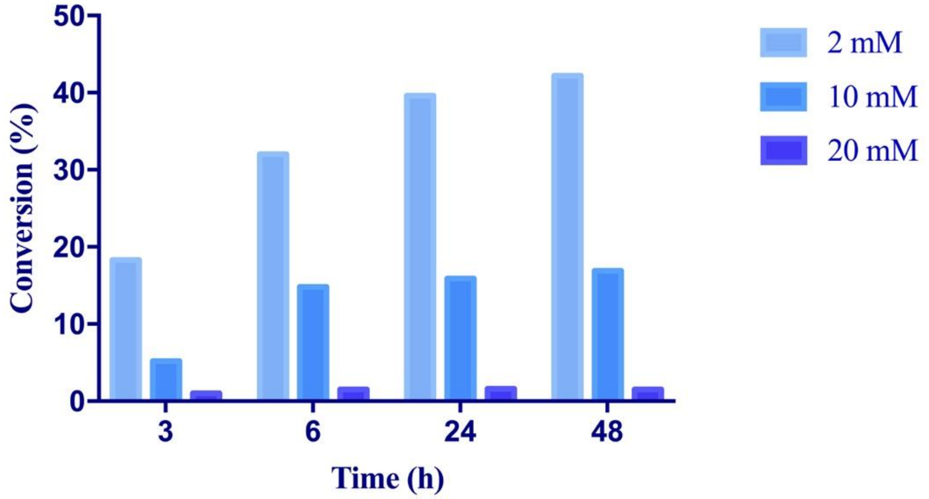

Figure S11. Time conversion profile for the ammonia addition onto *p*-CF_3-cinnamic acid **2l** using I460V *Pc*PAL.

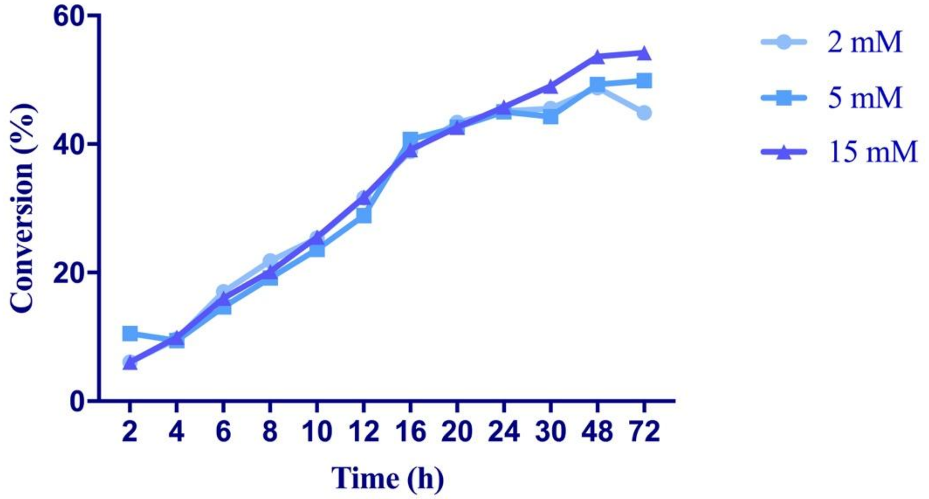

Figure S12. Time conversion profile for the ammonia elimination from *p*-CH_3-amino acid *rac*-**1c** using I460V *Pc*PAL.

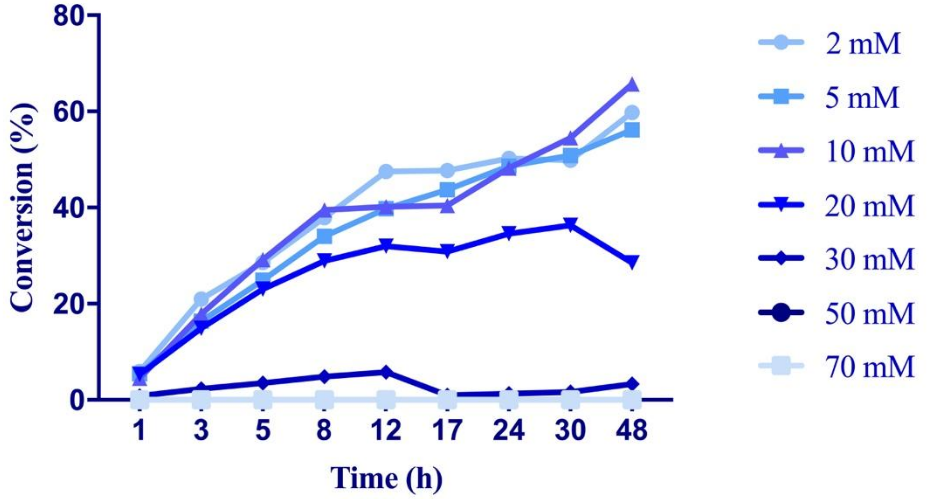

Figure S13. Time conversion profile for the ammonia elimination from *m*-CF_3-amino acid *rac*-**1k** using I460V *Pc*PAL.

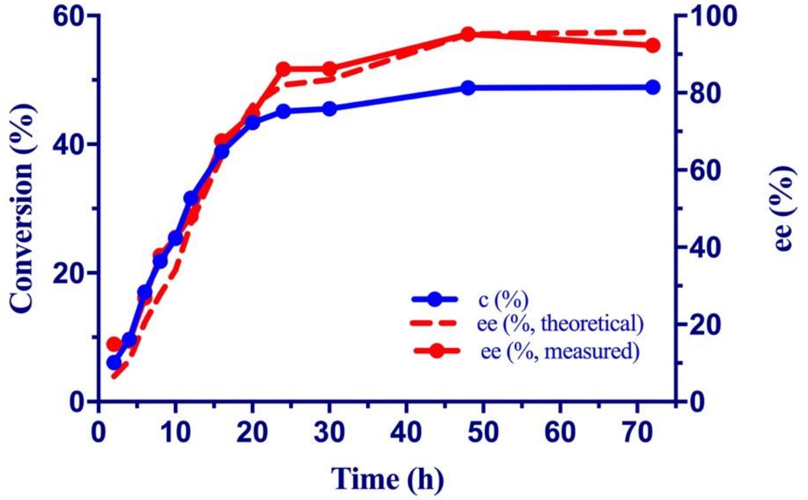

Figure S14. Conversion- and enantiomeric excess - time progression curve for the ammonia eliminations from *p*-CH_3-amino acid *rac*-**1c** using I460V *Pc*PAL and 2 mM substrate concentration..

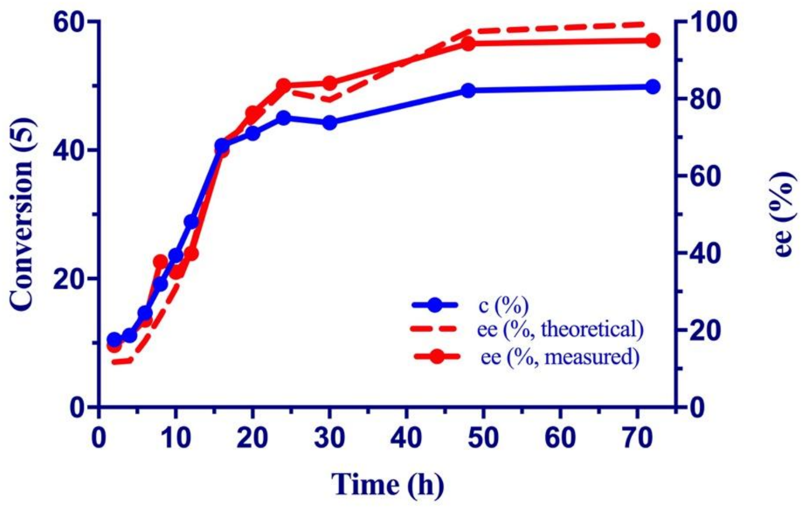

Figure S15. Conversion- and enantiomeric excess - time progression curve for the ammonia eliminations from *p*-CH_3-amino acid *rac*-**1c** using I460V *Pc*PAL and 5 mM substrate concentration.

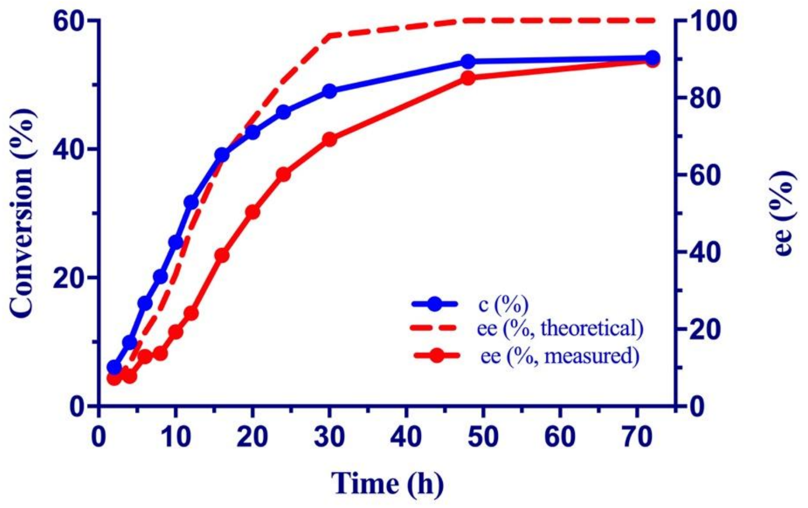

Figure S16. Conversion- and enantiomeric excess - time progression curve for the ammonia eliminations from *p*-CH_3-amino acid *rac*-**1c** using I460V *Pc*PAL and 15 mM substrate concentration.

3.4. HPLC chromatograms from the preparative scale biotransformations

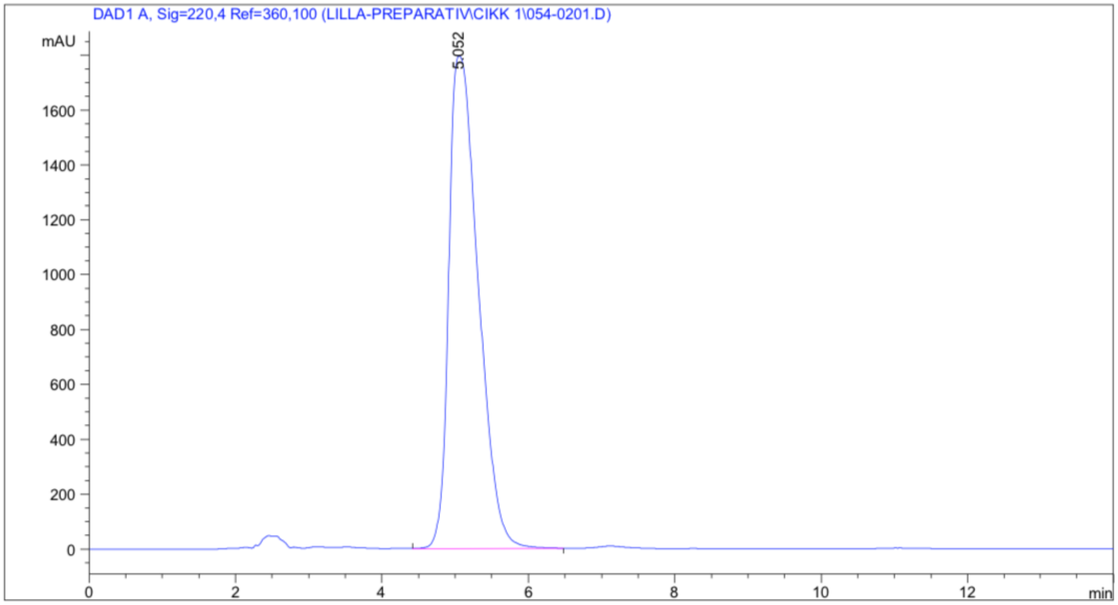

Figure S17. HPLC chromatogram of the reaction product L-**1e** from the L134A-*Pc*PAL catalyzed ammonia addition reaction of **2e** (ee>99%).

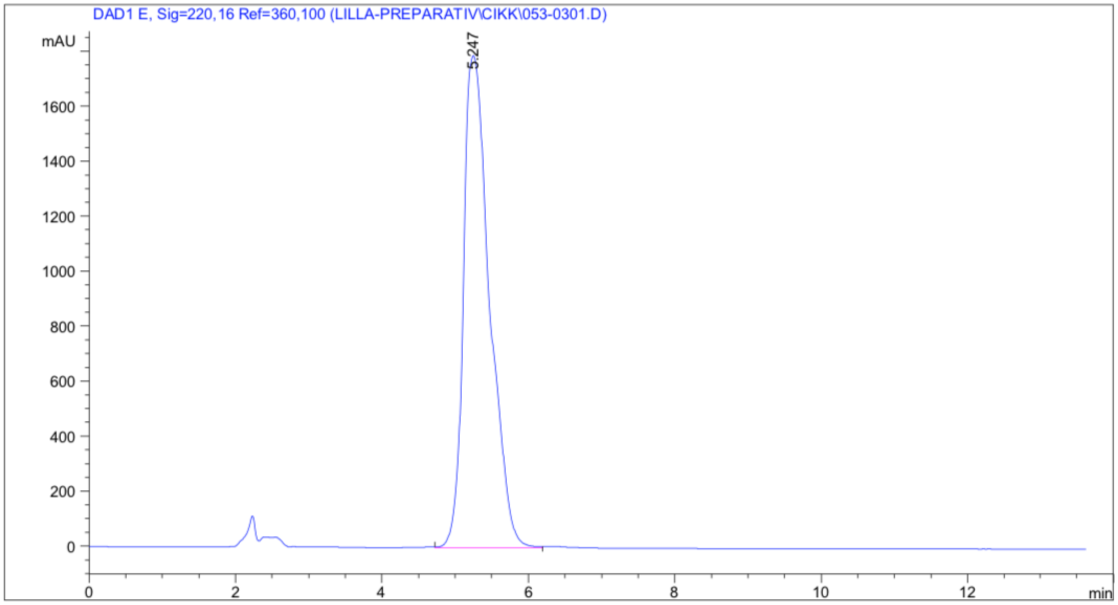

Figure S18. HPLC chromatogram of the reaction product L-**1i** from the I460V-*Pc*PAL catalyzed ammonia addition reaction of **2i** (ee>99%).

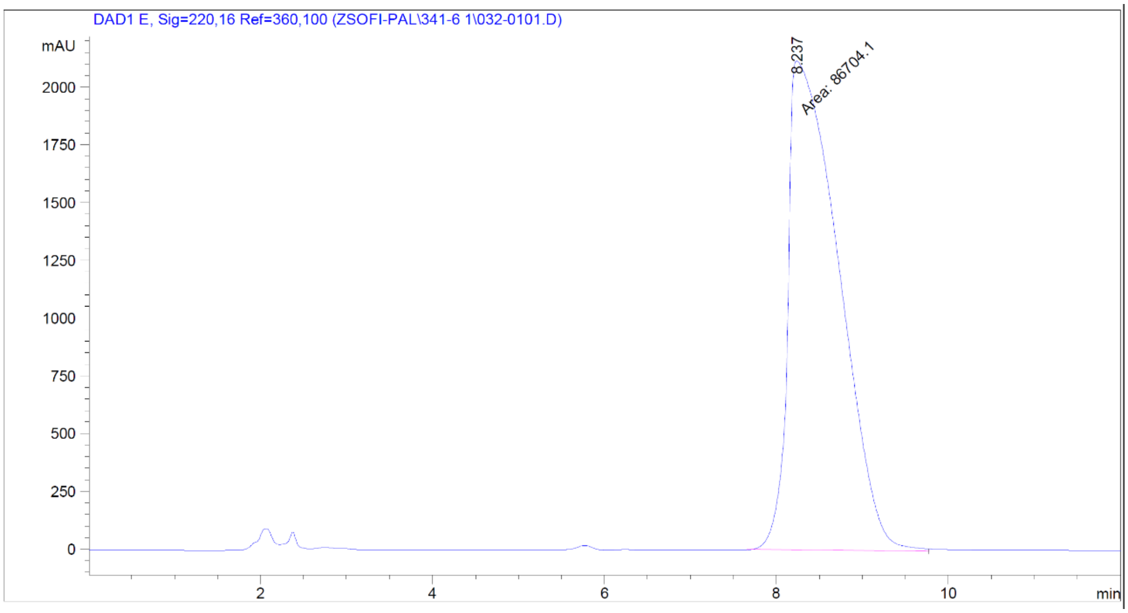

Figure S19. HPLC chromatogram of the reaction product L-**1k** from the I460V-*Pc*PAL catalyzed ammonia addition reaction of **2k** (ee>99%).

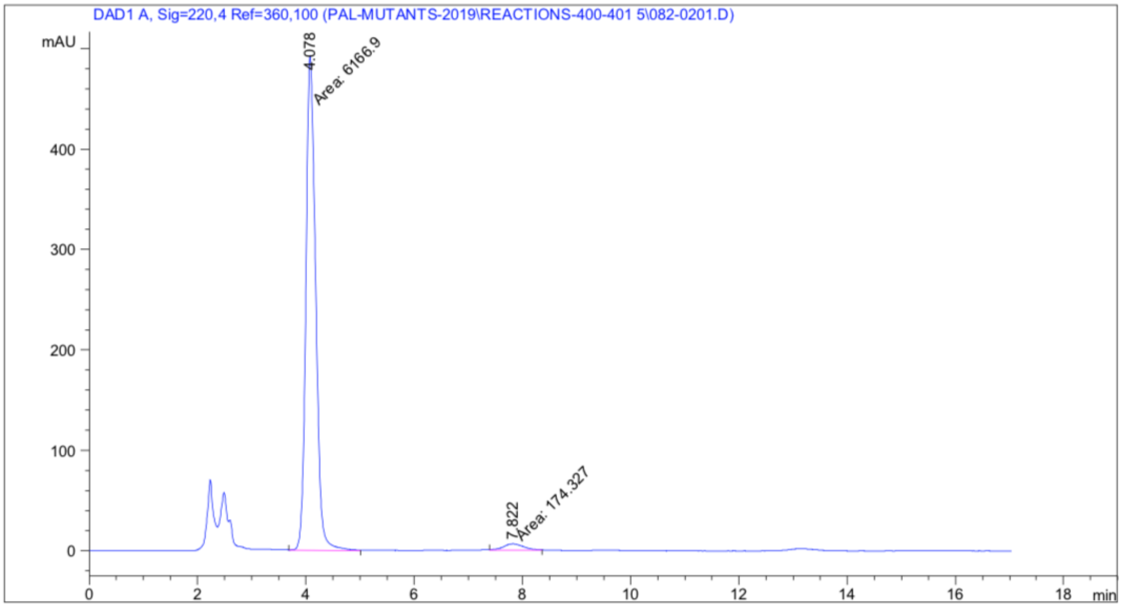

Figure S20. HPLC chromatogram of the isolated D-**1c** from the I460V-*Pc*PAL catalyzed ammonia elimination reaction of *rac*-**1c** (ee=95%).

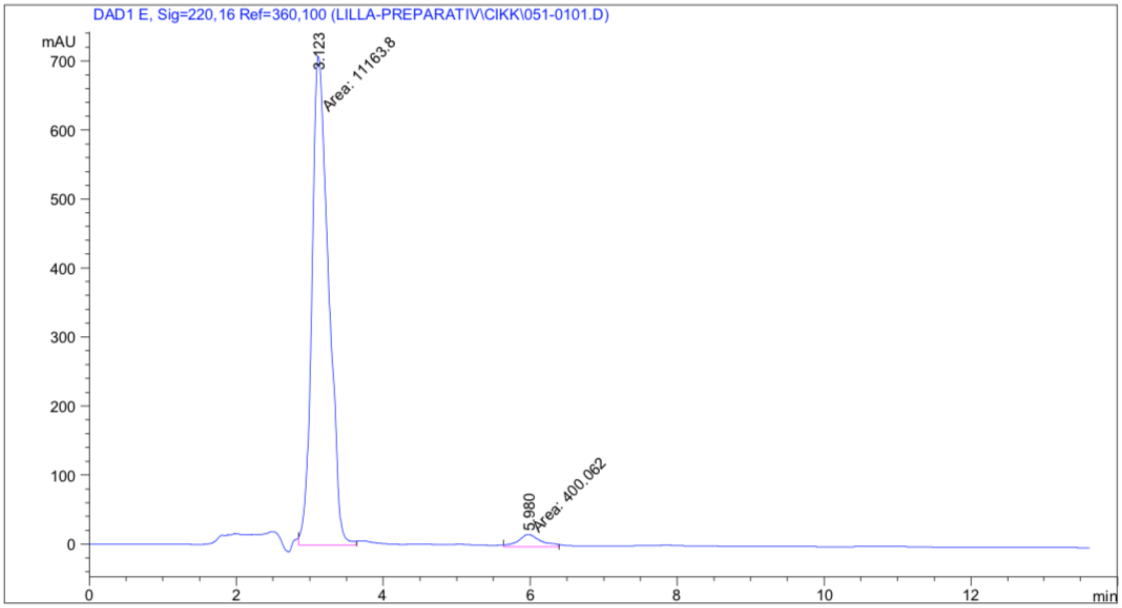

Figure S21. HPLC chromatogram of the isolated D-**1k** from the I460V-*Pc*PAL catalyzed ammonia elimination reaction of *rac*-**1k** (ee=93%).

3.5. Substrate inhibition in the ammonia addition reaction of *p*-CF_3-cinnamic acid 2l catalysed by I460V *Pc*PAL

The inhibitory effect of substrate in the ammonia addition reaction of *p*-CF_3-cinnamic acid **2l** catalysed by I460V *Pc*PAL was measured by monitoring the production of L-**1l** amino acid at 316 nm during 5 minutes using 5 µg of purified I460V *Pc*PAL and different concentration of **2l** (0.5-30 mM) in 6 M NH_4OH at pH 10 in a reaction volume of 1000 µL.

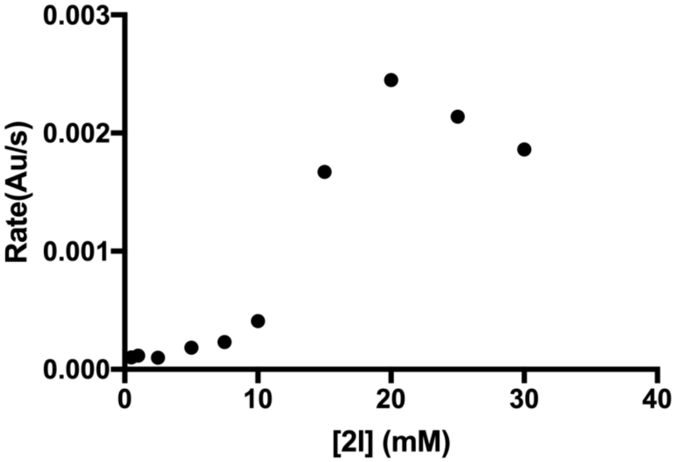

Figure S22. The inhibitory effect of increasing concentration of **2l** upon the reaction velocity of the ammonia addition reaction of *p*-CF_3-cinnamic acid **2l** catalysed by I460V *Pc*PAL.

3.5. Substrate inhibition in the ammonia elimination reaction of *m*-CF_3-amino acid *rac*-1k catalysed by I460V *Pc*PAL

The inhibitory effect of substrate in the ammonia elimination reaction of *m*-CF_3-amino acid *rac*-**1k** catalysed by I460V *Pc*PAL was measured by monitoring the production of cinnamic acid derivative **2k** at 285 nm during 5 minutes using 5 μg I460V *Pc*PAL and different concentration of *rac*-**1k** (0.1-50 mM) in 20 mM Tris buffer with 120 mM NaCl at pH 8.8 in a reaction volume of 200 μL.

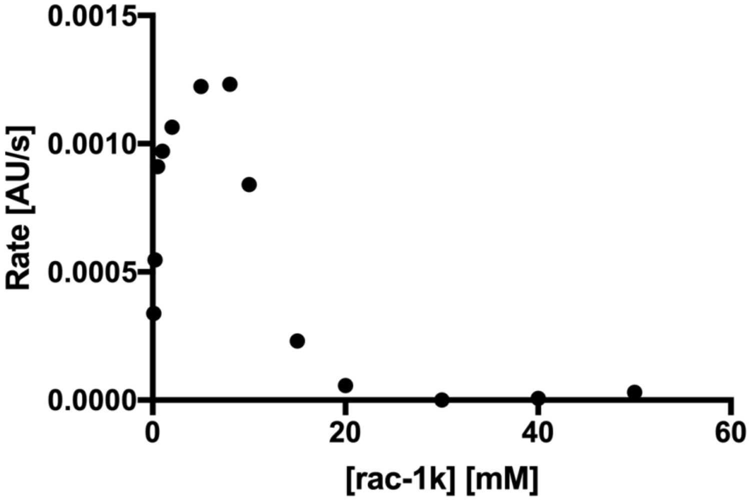

Figure S23. The inhibitory effect of increasing concentration on the reaction velocity in case of the ammonia elimination reaction of *m*-CF_3-amino acid *rac*-**1k** catalysed by I460V *Pc*PAL.

3.6. Product inhibition in the ammonia addition reaction of *m*-CH_3-cinnamic acid 2b and *p*-CH_3O-cinnamic acid 2f catalysed by I460V *Pc*PAL

The product inhibitory effect in the ammonia addition reactions of *m*-CH_3-cinnamic acid **2b** and *p*-CH_3O-cinnamic acid **2f** catalysed by L134A and I460V *Pc*PAL, respectively was measured by monitoring of the production of amino acid derivative L-**1b** respective L-**1f** in the presence of different concentrations of *rac*-**1b** and *rac*-**1f** (0-1.5 mM), using whole cells of L134A and I460V *Pc*PAL as biocatalyst. The reactions were performed in 6 M NH_4OH at pH 10 in a reaction volume of 1000 μL, using fixed, 1 mM substrate concentration of **2b** and **2f**.

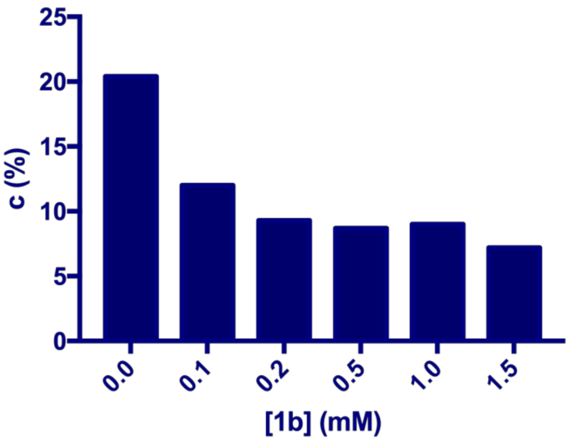

Figure S24. The influence of increasing the product (**1b**) concentration on the conversion values (after 12 h reaction times) of ammonia addition onto *m*-CH_3-cinnamic acid **2b**.

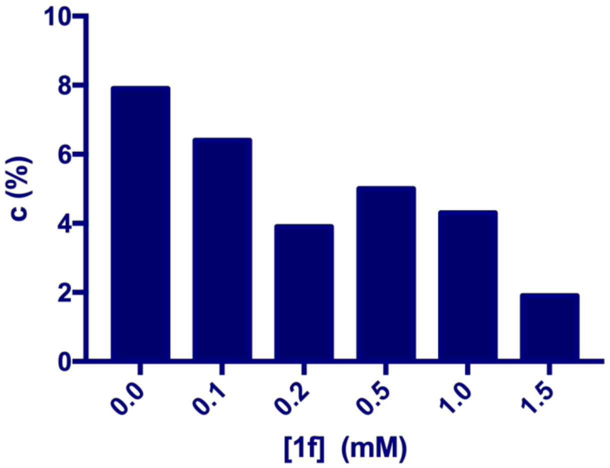

Figure S25. The influence of increasing the product (**1f**) concentration on the conversion values (after 12 h reaction times) of ammonia addition onto *p*-CH_3O-cinnamic acid **2f**.

Moreover, the product inhibitory effect was also analysed by kinetic measurements, by UV-monitoring the production of the amino acid derivative L-**1f** from the ammonia addition reaction onto **2f**, in presence of different concentrations of *rac*-**1f**. The reactions were monitored at 316 nm for 5 minutes, using 5 µg of purified I460V *Pc*PAL, 1 mM *p*-CH_3O-cinnamic acid **2f,** and different pconcentrations of *rac*-**1f**, ranging from 0-2 mM. The reactions were carried out in in 6 M NH_4OH, pH 10.0 in 1 mL reaction volume, at 30 ^{0}C.

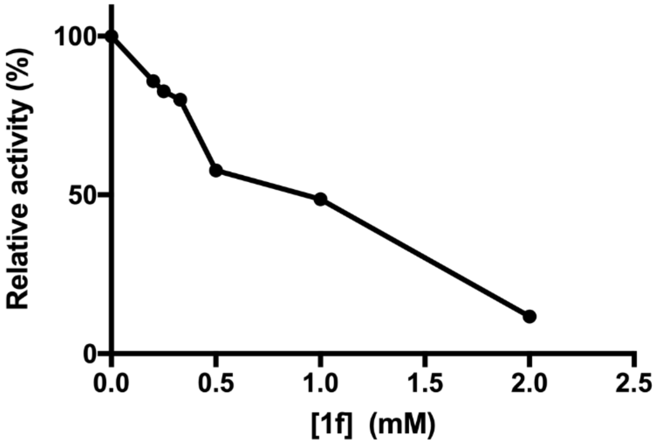

Figure S26. The inhibitory effect of increasing the product concentration of **1f** in case of the ammonia addition reaction of *p*-CH_3O-cinnamic acid **2f**, determined by kinetic measurements using purified I460V *Pc*PAL.

4. References

1. Nagy, E. Z. A. *et. al.* Mapping the hydrophobic substrate binding site of phenylalanine ammonia lyase from *Petroselinum crispum*. *ACS Catal.* **9**, 8825-8834 (2019).

2. Filip A. *et. al.* Tailored mutants of phenylalanine ammonia-lyase from *Petroselinum crispum* for the synthesis of bulky L- and D-arylalanines. *ChemCatChem*. **10**, 2627 – 2633 (2018).

3. Dima N. A. *et. al*. Expression and purification of recombinant phenylalanine ammonia-lyase from *Petroselinum crispum*, *Stud. Univ. Babes-Bolyai Ser. Chem.* 61, 21–34 (2016).
